# Multimodal profiling of pro-inflammatory protease activity identifies caspase-1 as a target for lung cancer interception

**DOI:** 10.1126/sciadv.adz4263

**Published:** 2026-08-14

**Authors:** Cathy S. Wang, Qian Zhong, Shih-Ting Wang, Carmen Martin-Alonso, Sofia Neaher, Sahil Patel, Tiziana Parisi, Jesse D. Kirkpatrick, Lecia V. Sequist, Tyler E. Jacks, Sangeeta N. Bhatia

**Affiliations:** ^1^ Department of Biological Engineering, Massachusetts Institute of Technology, Cambridge, MA 02139, USA.; ^2^ Koch Institute for Integrative Cancer Research, Massachusetts Institute of Technology, Cambridge, MA 02139, USA.; ^3^ Marble Center for Cancer Nanomedicine, Massachusetts Institute of Technology, Cambridge, MA 02139, USA.; ^4^ Institute of Medical Engineering and Science, Massachusetts Institute of Technology, Cambridge, MA 02139, USA.; ^5^ Division of Pulmonary and Critical Care, Department of Medicine, Massachusetts General Hospital, Boston, MA 02124, USA.; ^6^ Massachusetts General Hospital Cancer Center, Boston, MA 02114, USA.; ^7^ Department of Medicine, Massachusetts General Hospital and Harvard Medical School, Boston, MA 02115, USA.; ^8^ Department of Biology, Massachusetts Institute of Technology, Cambridge, MA 02139, USA.; ^9^ Ludwig Center at MIT’s Koch Institute for Integrative Cancer Research, Massachusetts Institute of Technology, Cambridge, MA 02139, USA.; ^10^ Wyss Institute for Biologically Inspired Engineering, Harvard University, Boston, MA 02215, USA.; ^11^ Howard Hughes Medical Institute, Chevy Chase, MD 20815, USA.

## Abstract

Systemic inhibition of interleukin-1b (IL-1b) has been shown to reduce the incidence of lung cancer in patients in years after treatment, but knowledge gaps surrounding its activation and role in the tumor microenvironment hinder cancer interception. We developed activity-based technologies to probe inflammation in early lung cancer and identified a translational target candidate. Probes sensitive to IL-1b–activating proteases were designed and applied to a murine model of inflammatory lung cancer, *Kras/Trp53*-mutant mice with SIINFEKL expression (KPS). Our nanosensors revealed elevated caspase-1 expression and activity in tumors, highlighting the importance of caspase-1 processing IL-1b during cancer development. We conducted a preclinical combination therapy trial by administering IL-1b blockade and caspase-1 inhibition. We observed significant reduction in lung cancer, including complete ablation of tumor incidence in nearly 20% of KPS mice. Our approach to understanding the interplay of protease activity and cytokine activation supports new strategies to mitigate inflammation and intercept lung cancer progression.

## INTRODUCTION

Lung cancer is the second most common cancer globally, as well as the leading cause of cancer death in both men and women in the United States (*1*). Despite advances in chemo- and immunotherapies, 5-year survival rates are still low due to late diagnosis. Cancer interception emerges as a crucial approach to detect and prevent cancer development. A promising indication for lung cancer interception was revealed by the CANTOS trial in 2017, in which systemic administration of interleukin-1b (IL-1b) antibody led to a significant reduction in lung cancer incidence and mortality in patients years later (*2*). However, when IL-1b blockade was applied to patients with late-stage non–small cell lung cancer (NSCLC) in subsequent CANOPY trials, very little improvement in survival was observed (*3*). This finding suggests that IL-1b inhibition might offer an opportunity for cancer interception with sufficiently early anti-inflammatory treatment. This possibility has the potential to drastically alter lung cancer outcomes, but to reach this goal, a greater understanding of the factors that influence IL-1b–associated inflammation pathways in early tumorigenesis is needed.

IL-1b is a particularly appealing pathway to target, due to its pro-inflammatory function in both acute and chronic inflammation, which have been shown to lead directly to lung cancer. IL-1b transcription is triggered in response to Toll-like receptor engagement, and IL-1b’s binding to IL-1Rs on various cell types activates the macrophage mitogen-activated protein kinase (MAPK) and nuclear factor κB (NF-κB) pathways, leading to inflammatory cytokine induction and immune cell recruitment (*4*, *5*). IL-1b is up-regulated in tumor and adjacent lung tissue in NSCLC, and high serum protein levels in patients with lung cancer have been linked to poor survival (*6*, *7*). Yuan *et al.* demonstrated that IL-1b antibody blockade decreased tumor burden in mice with KRAS-mutant lung adenocarcinoma (LUAD), and Hill *et al.* further showed reduced tumor formation in epidermal growth factor receptor (EGFR)–mutant mice during exposure (*8*, *9*). Other studies have observed that IL-1b can lead to activation of immunosuppressive cells including M2 macrophages and myeloid-derived stem cells, in addition to increased epithelial-to-mesenchymal transition and invasiveness of cancer cells (*10*, *11*). IL-1b can act via two pathways: inflammasome-dependent and inflammasome independent. In these two pathways, IL-1b is produced as an inactive pro-form by different cell types and can be activated by multiple different proteases (*12*). Thus, IL-1b’s pro-inflammatory functions, and more broadly, chronic inflammation–induced lung cancer, depend on these immune-related IL-1b–cleaving proteases (*13*). However, there is a lack of clarity regarding which proteases participate and how cleavage-mediated IL-1b activation influences local cell populations toward tumorigenesis. Uncovering the role of these proteases in chronic inflammation–induced lung cancer will lead to new approaches for targeted perturbation and resulting cancer interception.

In this study, we used an autochthonous murine model of LUAD, *Kras/Trp53*-mutant mice with SIINFEKL expression (KPS), to create an inflammatory environment eliciting tumor growth starting at 4 to 6 weeks postinduction. To investigate the involvement and impact of protease activity in the progression of disease, we leveraged different proteases’ ability to recognize and cleave unique amino acid sequence motifs and designed a library of 124 fluorescence resonance energy transfer (FRET) substrates that included motifs for interleukin-processing proteases. After down-selection screening with recombinant proteases, 20 substrates were prioritized to create in vivo activity-based nanosensors (ABNs). ABNs, which have previously been used to track protease activity changes for disease diagnosis, can be used to profile which proteases are active during disease progression and measure changes in the presence or absence of treatments (*14*–*16*). This panel of ABNs was used to monitor the responses of KPS mice with early-stage tumors to weekly administration of IL-1b and programmed cell death protein 1 (PD-1) blockade. Our studies revealed that treated mice exhibited a reduction in caspase-1 activity, an enzyme known for its role in processing IL-1b (*17*). We found robust localization of caspase-1 activity to early-developing lung tumor nodules by applying a third peptide-based technology, an activity-based zymography probe, that fluorescently “paints” tissues that exhibit protease activity (*18*). On the basis of these findings, we hypothesized that caspase-1 activity contributes to the production of active IL-1b in early LUAD. As such, using a clinically safe small molecule inhibitor of caspase-1, we studied the effect of caspase-1 inhibition, alone and in combination with IL-1b blockade, in vivo. Caspase-1 inhibition resulted in a significant reduction in cancer progression in KPS mice, and a repeatable fraction of combination-treated mice had no detectable tumors. Collectively, we demonstrate how activity-based nanoscale technologies help to elucidate the inflammatory microenvironment in early cancer development and find a therapeutic target for lung cancer interception.

## RESULTS

### Peptide substrates were nominated for cleavage by IL-1b–activating proteases

**Fig. 1.**
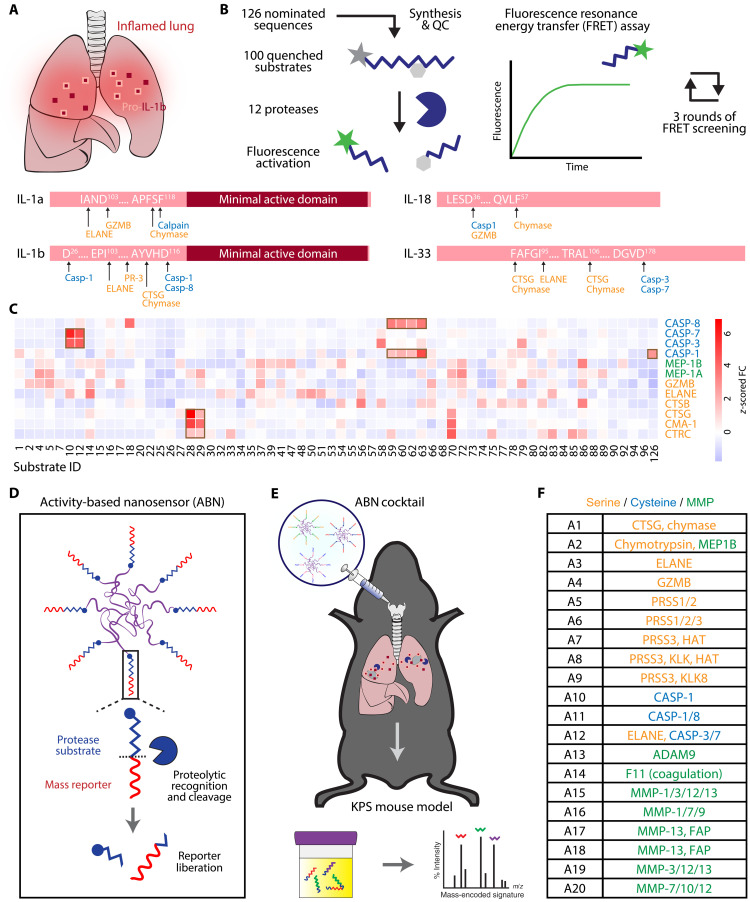
Interleukin-associated protease substrates were designed and screened to develop an in vivo sensor panel. (**A**) Schematic of inflamed lung with high expression of IL-1b in pro-form and cleavage sites of associated interleukins (*16*). (**B**) Schematic of FRET protease substrate screening. A total of 100 amino acid sequences with fluorophore-quencher pairs were designed or nominated from previous lung cancer diagnostic panels and incubated with 14 proteases across three rounds of FRET cleavage assays, where fluorescence increases over time with cleavage. QC, quality control. (**C**) Heatmap of *z*-scored cleavage fold change (FC) from *n* = 3 technical replicates in a representative FRET screen. Brown boxes indicate examples of strong cleavage trends for select probes. (**D**) Schematic of ABN cleavage by local proteases in vivo. (**E**) Intratracheal administration and mass spectrometry readout shows cleaved ABN reporters in urine from mice. *m/z*, mass/charge ratio. (**F**) List of ABN probes and corresponding target protease selected for in vivo administration.

Protease dysregulation has been known to play a role in oncogenic processes such as promoting growth, angiogenesis, and inflammation (*19*). Protease activity can be detected in vitro and in vivo through measuring fluorescence and mass-encoded readouts; these readouts are the result of cleavage of amino acid sequence substrates that are designed to be recognized by target proteases (*14*, *15*, *20*). As the cleavage and resulting activation of IL-1b in inflammation is also governed by proteases, we sought to monitor these upstream activators to identify potential changes that contribute to inflammation-associated lung cancer (Fig. 1A). We designed a library of 124 substrates consisting of 8 to 13 amino acid sequences with motifs that would be recognized by proteases that cleave IL-1b and related interleukins (Fig. 1B and table S1) (*19*, *21*, *22*). Sequences that were successfully synthesized with a fluorophore-quencher pair were used in several rounds of FRET screening. Fluorescence was only detected when cleavage of the substrates separated the quencher from the fluorophore. We assayed FRET substrates against recombinant proteases of interest to determine the specificity and efficacy with which each protease cleaves a given substrate (fig. S1). We found various substrates that were recognized and cleaved effectively by at least one of the proteases. Because of the known promiscuity of proteases, some substrates such as #28, #29, and #70 were cleaved efficiently by multiple proteases of the same class (*20*).

For dynamic and longitudinal in vivo sensing of these proteases, we converted substrates that were efficiently cleaved by our proteases of interest into linkers for ABNs. To form these nanoparticles, polyethylene glycol (PEG) scaffolds are conjugated with unique mass-encoded tags via different cleavable substrate linkers (Fig. 1D) (*23*). Upon intratracheal delivery of ABNs, local proteases in the lung can recognize and cleave specific amino acid sequence motifs, which releases the mass reporters into circulation for renal concentration and urinary clearance. Mass spectrometry of the collected urine samples reveals the amount and identity of the reporter tags, and by proxy, the protease-cleaved substrates (Fig. 1E). Our multiplexed panel of ABNs consisted of 20 substrates of previously validated lung cancer targets, as well as hits from screening recombinant IL-1b–activating proteases, designed to detect changes in protease activity in the lung with IL-1b–associated perturbation (Fig. 1F and table S2) (*14*).

### IL-1b blockade in inflammatory lung cancer model results in differential protease cleavage

**Fig. 2.**
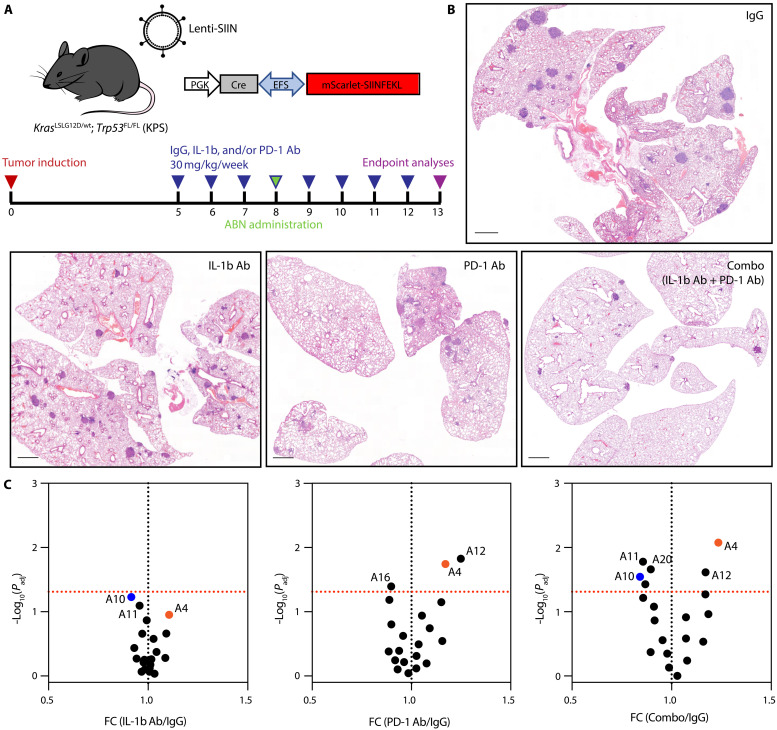
Protease activity differentiates treated versus untreated KPS LUAD. (**A**) KP mice were injected intratracheally with lentivirus encoding for Cre recombinase and the model antigen SIINFEKL (Lenti-SIIN; red triangle) to develop the inflamed “KPS” model. Weekly antibody (Ab) treatments were started 5 weeks post–tumor induction (black triangles). After 3 weeks of treatment, a panel of ABNs was administered and cleaved reporters were collected in urine (green triangle). After 8 weeks of treatment, mice were euthanized, and tissues were collected for endpoint analyses (purple triangle). *N* = 11 to 15 mice in each treatment group. (**B**) Histology of fixed lung tissue after treatment shows tumor burdens under different therapeutic conditions (IgG control antibody, IL-1b antibody, PD-1 antibody, or combination of IL-1b and PD-1 antibodies). Scale bar, 1 mm. (**C**) Volcano plots show the average cleavage fold change (*n* = 11 to 15 mice) for each substrate in the ABN panel after 3 weeks of treatment compared to the IgG control group. Student’s *t* test with Bonferroni correction was performed for each substrate’s *P*_adj_. The A4 (orange) and A10 (blue) substrates were found to be efficiently cleaved by granzyme B and caspase-1, respectively.

The ABNs were applied to the LUAD KPS murine model, where lung tumors were induced in *Kras/Trp53*-mutant mice by intratracheal injection of lentivirus encoding for both Cre recombinase and SIINFEKL. The expression of the SIINFEKL tumor antigen in KPS mice creates a more immunogenic lung environment in which the tumors grow, as shown through IL-1b expression in tumor nodules as early as 6 weeks (fig. S2) (*24*, *25*). In congruence with prior studies, we adopted a dosing regimen of intraperitoneal administration with either control immunoglobulin G (IgG) antibody, IL-1b antibody, PD-1 antibody, or combined IL-1b and PD-1 antibodies to KPS mice, starting at early-stage disease, 5 weeks after tumor inoculation (Fig. 2A) (*8*). We observed that introducing IL-1b blockade at these early stages reduced tumor formation, consistent with previous studies in various LUAD models (Fig. 2B) (*8*, *9*). We then wanted to characterize the pattern of protease activity in the early inflammatory environment and detect when and how activation of the IL-1b pathway might play a role in cancer progression.

Using the same dosing scheme, we administered the 20-substrate panel of ABNs 3 weeks after initiating treatment protocols (8 weeks after inducing tumorigenesis) and collected urine to assess substrate cleavage. We found several differentially cleaved substrates at this time point (Fig. 2C). In particular, A10, sensitive to caspase-1 activity, exhibited the greatest reduction in cleavage fold change after IL-1b antibody treatment, relative to IgG control-treated tumor-bearing mice (fig. S3). With PD-1 antibody treatment, more sensor release was observed for the neutrophil elastase (ELANE) (A12) and granzyme B (A4) substrates, which is consistent with ongoing antitumor cytotoxic immunity in these treated mice (fig. S4) (*26*). In the case of combination therapy, we saw both effects: increased ELANE and granzyme B activity in addition to decreased caspase-1 activity (A10 and A11).

### Immunofluorescence and AZP staining show IL-1b–associated proteases in KPS tissue

**Fig. 3.**
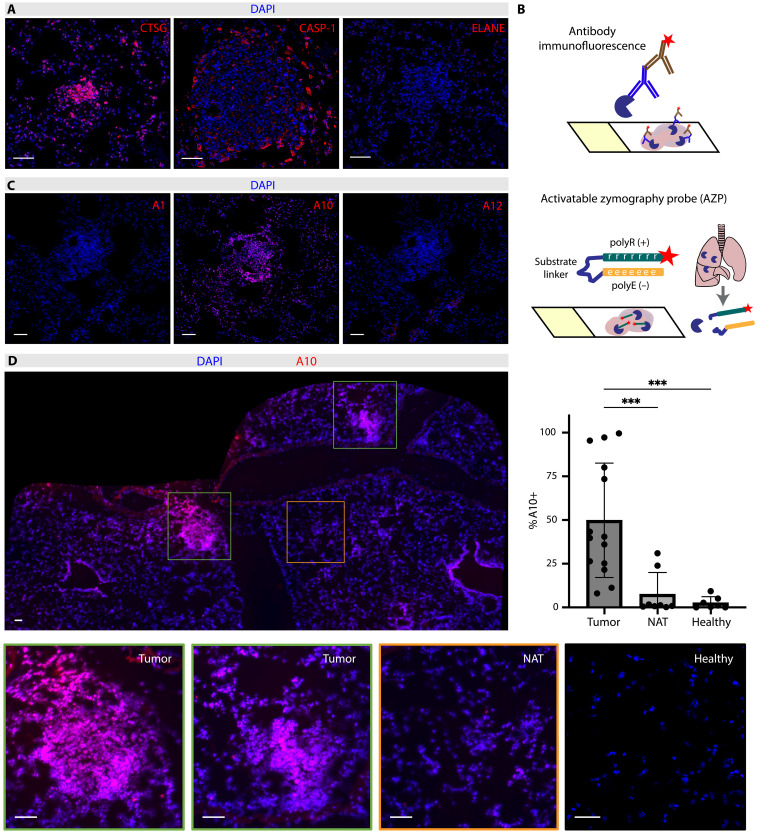
Caspase-1 is highly expressed and highlights tumors in an activity-dependent manner. (**A**) Immunofluorescence staining of protease protein expression (red): CTSG, caspase-1, and ELANE, in 6-week KPS tumors. All sections counterstained with 4′,6-diamidino-2-phenylindole (DAPI) (blue). Scale bars, 50 μm. (**B**) Schematic of antibody versus AZP staining on frozen tissue sections. Protease cleavable substrates can be converted to AZPs that show cleavage locally on tissue. AZPs consist of a negatively charged reporter masked by a peptide substrate sequence that prevents it from binding to tissue until cleaved by localized target proteases. (**C**) Binding of activated AZPs (red) cleavable by CTSG (A1), caspase-1 (A10), and ELANE (A12) in lung tumor tissue. All sections counterstained with DAPI (blue). Scale bars, 50 μm. (**D**) Comparison of A10 AZP staining in 7-week KPS lung sections between tumors and NAT or healthy tissue. All sections counterstained with DAPI (blue). Percentage of tissue regions that were positively stained for A10 was quantified (*n* = 7 to 14 tissue regions). One-way ANOVA with Tukey’s multiple comparison test was performed (*P* < 0.05). Scale bars, 25 μm.

In addition to using ABNs to measure protease activity in vivo, we sought to evaluate the spatial abundance of IL-1b–associated proteases in the KPS mice. We compared immunofluorescence staining for caspase-1, cathepsin G (CTSG), and ELANE, which are known to be upstream mediators of IL-1b activation, in KPS lung tissue sections over time. We found high levels of caspase-1 and CTSG protein, as well as minimal expression of ELANE, in tumors that developed 6 weeks postinduction, and this pattern persisted through later stages (Fig. 3A and fig. S5). As expression does not necessitate enzymatic activity, we turned to activatable zymography probes (AZPs) as a tool to localize the observed protease cleavage activity within the sections (Fig. 3B). AZPs consist of a fluorophore-labeled, positively charged domain linked to a masking agent via a protease-cleavable substrate sequence (*18*). When this substrate is cleaved by local protease activity, the positively charged fluorescent fragment can bind to negatively charged tissue in the vicinity. The target substrates were chosen from our FRET screen and ABN panel, in which we had identified specifically and efficiently cleaved substrates for each protease of interest. A1, A10, and A12 are cleaved robustly by recombinant CTSG, caspase-1, and ELANE, respectively (Fig. 1C and fig. S4). AZP staining in early-stage KPS tumors showed high A10 signal but minimal cleavage-specific A1 and A12 signal, as indicated by the changes in fluorescence signal with the coincubation of broad-spectrum inhibitors that block enzymatic cleavage (Fig. 3C, fig. S6, and table S3). These findings were consistent with the absence of immunofluorescence-marked expression of ELANE in tumors, as well as the lack of CTSG activity among the in vivo nanosensors. With further exploration of A10, we observed a robust fluorescence AZP signal that highlighted tumors in the KPS lung tissue. In contrast, significantly less A10 staining was detected in both normal adjacent tissue (NAT) and in healthy tissue samples, indicating tumor-specific cleavage and binding of the probe (Fig. 3D). Collectively, the detection trends of the A10 sensor were consistent with the hypothesis that caspase-1 activity is present in early-stage tumors and potentially contributes to cancer progression in untreated KPS mice.

### A10 highlights caspase-1 activity in lung tumors

**Fig. 4.**
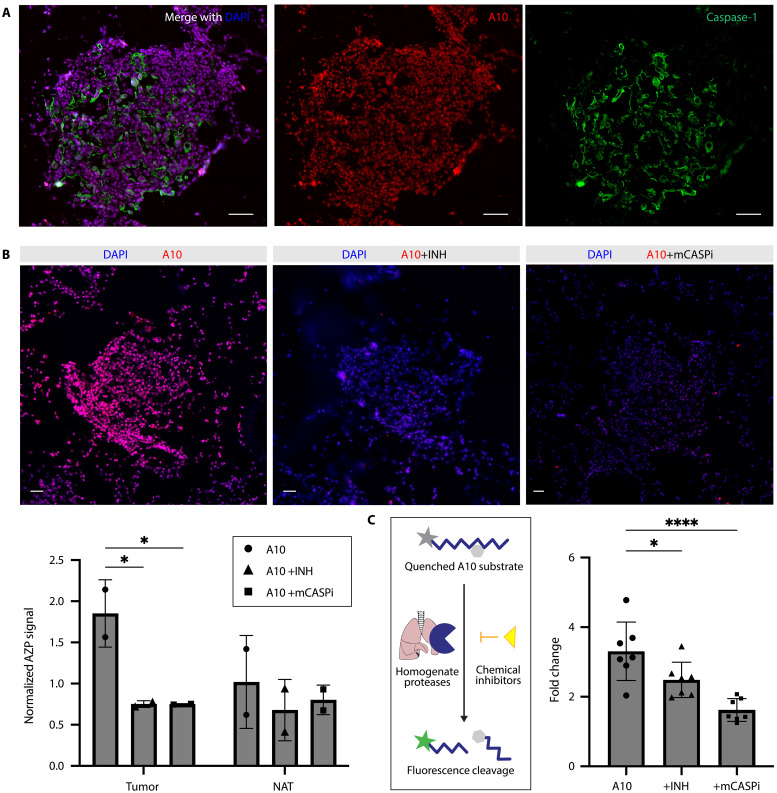
A10 AZP cleavage is abrogated by caspase-1 inhibition. (**A**) Costaining of caspase-1 with A10 AZP in 7-week KPS tumors. Scale bars, 50 μm. (**B**) A10 AZP-stained 7-week KPS lung sections were incubated with a broad-spectrum inhibitor (INH) or metabolized caspase-1–specific inhibitor (mCASPi). All sections counterstained with DAPI (blue). Normalized A10 AZP signals were quantified and compared using the one-way ANOVA with Tukey’s multiple comparison test (*n* = 2 mice, *P* < 0.05). Scale bars, 25 μm. (**C**) Eleven-week KPS lung homogenates were incubated with the A10 FRET probe, INH, and/or mCASPi, where fold changes indicate increase in fluorescence from cleavage after 120 min. The one-way ANOVA with Tukey’s multiple comparison test was performed (*n* = 7 mice, *P* < 0.05).

Costaining tumor sections with the A10 AZP and a caspase-1 antibody showed protein expression colocalizes with sensor cleavage in both early- and late-stage KPS tumors (Fig. 4A and fig. S7). The A10 activity-based probe’s staining pattern does not exactly overlap with the antibody’s immunofluorescence binding, likely due to the limited resolution of probe binding. In this case, upon activation by enzymatic cleavage, the AZP’s positive probe fraction is liberated and seeks to bind to nearby, negatively charged nuclei as the strongest electrostatic interaction. This outcome results in regional localization, but a lower cellular staining resolution compared to antibodies, for which the fluorescent label binds directly to a specific protein epitope. When the tissue samples exposed to the A10 AZP were coincubated with a broad-spectrum protease inhibitor or a metabolized caspase-1–specific inhibitor, VRT-043198, we observed a significant reduction in fluorescence labeling of the tumors (Fig. 4B). This inhibition-dependent decrease in tumor staining suggested that caspase-1 was actively contributing to local tissue cleavage of A10. As caspase-1 activation is predominantly mediated by inflammasome assembly, we showed that there was high expression of the key adaptor protein, apoptosis-associated speck-like protein containing a caspase-recruitment domain (ASC), in early-stage KPS tumors (fig. S8) (*27*). We further validated the role of caspase-1 in cleaving the A10 substrate using lung homogenates from late-stage KPS mice in a FRET inhibitor assay (Fig. 4C and fig. S9). We found that most of the cleavage signal produced by the tumor-bearing lung tissue samples was due to caspase-1, given that the fold change in fluorescence signal was significantly lower when tumor homogenate samples were incubated with the caspase-1–specific inhibitor.

### Human LUAD samples exhibit high caspase-1 expression and activity

Beyond the KPS model, we explored the translational relevance of caspase-1 by examining human LUAD samples. Although a more statistically powered study would be needed to confirm significant differences, immunohistochemical staining of caspase-1 in sections of three human lung biopsies showed elevated numbers of caspase-1–positive cells in tumor areas, relative to adjacent healthy areas (fig. S10). We also tested bronchioalveolar lavage fluid (BALF), which is a common form of clinical samples that capture interstitial cells and proteins from the lungs. We hypothesized that caspase-1 would be released and active in this compartment in samples from patients with LUAD. BALF samples were procured from healthy smokers with no detectable cancer, as well as from smokers who were patients with stage I/II LUAD. The BALF samples, normalized by total protein concentration, were assessed for caspase-1 content via enzyme-linked immunosorbent assay (ELISA). There was no detectable caspase-1 in healthy BALF, in contrast to varying higher levels of caspase-1 in cancer samples (fig. S11A). To evaluate caspase-1 activity, we incubated the BALF samples with quenched luciferase-conjugated caspase-1 substrates, where high luminescence indicated that active caspase-1 from the samples had recognized and cleaved the substrate. BALF samples from patients with LUAD had significantly higher luminescence signals (fig. S11B). Although all the human BALF samples, from both healthy donors and patients with lung cancer, come from smoking backgrounds, we found greater caspase-1 expression and activity only in the patients with cancer, suggesting cancer-specific involvement of caspase-1, rather than due to generally inflamed environments.

### Combined blockade of IL-1b and caspase-1 significantly reduces lung tumor formation

**Fig. 5.**
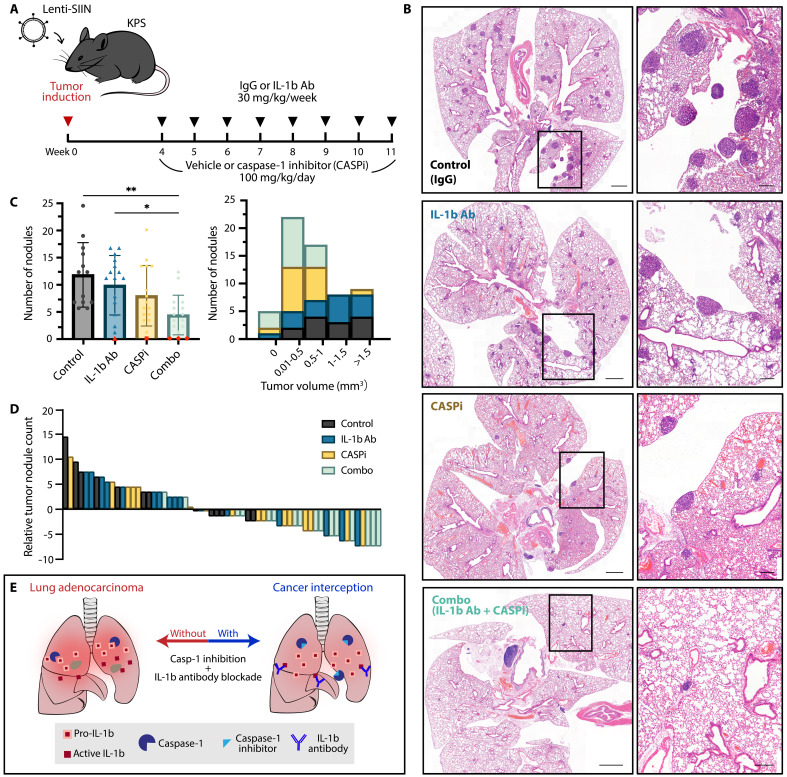
Combined caspase-1 inhibition and IL-1b blockade reduces KPS lung tumor burden. (**A**) KPS mice were induced with lentivirus encoding for Cre recombinase and the model antigen SIINFEKL. Weekly antibody treatments (30 mg/kg) and daily oral gavage of the caspase-1 inhibitor belnacasan (CASPi; 100 mg/kg) were started 4 weeks post–tumor induction. (**B**) Histology for representative lungs from each treatment group (scale bars, 1 mm) with enlarged insets (scale bars, 250 μm). (**C**) Tumor burden volume and (**D**) number of nodules were quantified from micro-CT images (*n* = 13 to 16 mice combined from two independent cohorts). Mice with no detectable nodules are marked with red circles (C, left) or listed as having 0 tumor volume (C, right). The one-way ANOVA with Tukey’s multiple comparison test was performed (*P* < 0.05). (**E**) Schematic of lung cancer interception leveraging combined caspase-1 inhibition and IL-1b blockade to reduce inflammation.

As caspase-1 expression and activity have been shown to be elevated in both murine and human tumor samples, we hypothesized that caspase-1 may act as an upstream activator of protumor inflammation and that we could therapeutically perturb it to prevent tumorigenesis. We explored the use of a small molecule caspase-1 inhibitor, belnacasan, at a daily oral gavage dose of 100 mg/kg (*28*). We combined belnacasan with the IL-1b antibody and compared it with control IgG antibody or IL-1b antibody alone (Fig. 5A). We started administering the candidate therapeutics to KPS mice early in cancer development, 4 weeks after tumor inoculation. After 7 weeks of treatment, we detected significantly fewer tumor nodules when belnacasan was administered, and this improvement was even greater when combined with IL-1b antibody blockade, as shown by both lung cross-sectional histology and volumetric micro–computed tomography (micro-CT) quantification (Fig. 5, B to D, and fig. S12). Moreover, the total tumor burden was also reduced in the caspase-1–inhibited cohort, and the individual tumor nodules that were detected in treated mice were much smaller. Notably, 18% of combination-treated KPS mice bore no visible tumors, highlighting a marked example of lung tumor interception. Immunohistochemical staining of active IL-1b in sections of treated lungs further shows decreased IL-1b and local reduction of inflammation (fig. S13). As caspase-1 can cleave and activate other proteins, it is also likely that, in addition to IL-1b, immune factors, like IL-18 and caspase-7, could be affected due to caspase-1 inhibition (*29*). Although IL-1b antibody alone lacked generalizable therapeutic efficacy, as seen by the wide distribution spread in tumor burden, the combined blockade with caspase-1 inhibition more comprehensively blocked both the activation and pro-inflammatory function of IL-1b, leading to a drastically improved response rate (Fig. 5E).

## DISCUSSION

Here, we integrated activity-based nanotechnologies across hierarchical scales to probe IL-1b–associated proteolytic activity and its contribution to the progression of inflammatory LUAD. We uncovered caspase-1 as a highly expressed and active upstream regulator of IL-1b in KPS tumors and developed a therapeutic approach that combined IL-1b blockade with caspase-1 inhibition in early-stage LUAD to improve cancer outcomes.

IL-1b antibody (canakinumab) treatment was reported to reduce the overall cancer mortality and incidence of lung cancer in patients in the CANTOS trial, but there was little improvement in survival when a similar regimen was applied for advanced or metastatic NSCLC (*2*). These observations suggest that knowledge gaps surrounding IL-1b activation and its role in the tumor microenvironment (TME) are hindering the development of a more effective therapeutic approach in LUAD. We were able to use a representative inflammatory LUAD murine model to make advances in understanding IL-1b activation and its role in the TME. Leveraging potential field cancerization in the lungs, our ABN platform captured and monitored dysregulated protease activity in the local lung environment in vivo (*16*, *23*, *30*). We designed a panel using substrates that were previously identified to differentiate lung cancer from healthy mice and new substrates that we screened against upstream activators of IL-1b (*14*, *17*). We observed increased cleavage of a substrate susceptible to granzyme B activity upon treatment, consistent with its role as a mediator of cytotoxic lymphocyte activity (*31*). In contrast, among all the proteases we probed that are involved in IL-1b activation, caspase-1 activity decreased most significantly with treatment and tumor cell death, thus appearing to contribute the most to the cancer state. Consistent with the high expression of the inflammasome component ASC in early-stage KPS lungs, caspase-1’s elevated activity exerts a dichotomous influence in cancer through the inflammasome pathway: Pyroptosis can kill tumor cells, but IL-1b activation can support tumor growth and immune escape during chronic inflammation (*32*, *33*).

Although we did not pinpoint the specific cell source for caspase-1 production and activation in LUAD progression, we found colocalization between caspase-1 expression and epithelial cells in early-stage KPS tumors (fig. S14). There is also a generally high infiltration of immune cells in lungs of early-stage LUAD, including macrophages (15%) more than T cells (4 to 6%) and neutrophils (2 to 3%), and we observe similar staining distributions between IL-1b and the macrophage marker F4/80 in KPS tumors (figs. S14 and S15) (*34*). IL-1b and caspase-1 may be produced by different cell types, and the cellular contribution may change with disease progression as the infiltrating populations change. Cellular analysis, such as with single-cell RNA sequencing, for LUAD might be able to elucidate the mechanistic caspase-1–associated genetic programs in these different cell populations that result in tumor development. The balance of immunosuppressive and immune-activating populations would also merit further exploration, especially with LUAD progression that might lead to shifting roles from different cell types.

In addition to LUAD, inflammation from IL-1b signaling is also implicated in hematological malignancies and cardiovascular diseases (CVDs). Enriched levels of inflammasome components that activate caspase-1, as well as IL-1b, were found in macrophages in atherosclerotic CVD (*35*, *36*). Accumulation of IL-1b+ macrophages in tumors have been found to contribute to TME remodeling, metastasis, and therapeutic resistance (*37*). Tumor-infiltrating clonal hematopoiesis (TI-CH), most strongly predicted by TET2 or DNMT3A mutations, has also been found to increase the risk of NSCLC-associated death by remodeling the tumor immune microenvironment with enhancing monocyte migration (*38*, *39*). Mechanistically, TET2-deficient cells generate more inflammation by increasing inflammasome-mediated IL-1b secretion, highlighting patients with these mutations as a potential population that could benefit from our findings (*40*).

To more robustly block the protumorigenic IL-1b pathway via the upstream caspase-1, we proposed a therapeutic strategy combining IL-1b antibody and caspase-1 inhibitor, belnacasan. Belnacasan, or VX-765, is an oral prodrug found to reduce immune activation and delay pathology in inflammatory diseases (*41*). It has been used in clinical trials for psoriasis, epilepsy, and COVID-19 (NCT01048255, NCT00205465, NCT01501383, and NCT05164120). Although it was well tolerated and safe for administration in humans, it lacked therapeutic efficacy in these prior indications. Cancer applications of belnacasan are still being explored; one recent example includes its use in mice to improve sensitivity to immune checkpoint blockade in triple-negative breast cancer xenografts (*42*). In this study, we demonstrated that belnacasan could be repurposed for lung cancer interception. When applied at an early stage, caspase-1 inhibition alone significantly reduced tumor burden in KPS mice. Outcomes were reliably and further improved with the combination of IL-1b blockade, as indicated by the lower count and smaller size distribution of detected tumor nodules. This interceptive impact was achieved potentially through both reducing the activation of IL-1b and blocking the downstream functions of any active IL-1b. Although we were able to identify caspase-1 activity as an early carcinogenic event and therapeutic target in LUAD, future work to understand how other inflammatory factors change over time with progression would help determine the stage of intervention and patient stratification strategies to bring this finding closer to human application (*43*). For example, patients who have had a smoking history or frequent exposure to air pollutants would be at higher risk for developing lung cancer and could be candidates for this early therapeutic strategy. Developing clinical biomarkers, such as profiling caspase-1 activity in BALF or sequencing for TI-CH, could be a way to predict benefit in patient subpopulations. Furthermore, investigating the antigen-specific immune response from this combination treatment will be enlightening to understand any resulting adaptive immunity and long-term memory. Given inflammation’s far-reaching role in various other diseases, such as ulcerative colitis and rheumatoid arthritis, combination therapies that combine caspase-1 inhibition and a more disease-specific target may also be promising to explore more broadly.

Our study investigated and monitored IL-1b–associated protease dysfunction to seek mechanistic insights into early inflammatory lung cancer. The spatial and longitudinal characterization of proteases in the TME through various nanoscale modalities shed light on protumorigenic features of the lung TME as potential translational targets for cancer interception, amidst the cross-talk of inflammation and tumorigenesis. Combination therapy of IL-1b blockade with caspase-1 inhibition in early LUAD showed significant reduction in tumor burden, suggesting a potential path to cancer interception for at-risk patients with prolonged exposure to smoke or other air pollutants. We envision that early administration of this treatment in high-risk patient populations could transform the lung cancer interception landscape. More broadly, our activity-based nanotechnologies serve as a widely applicable way to probe and better find disease biology.

## MATERIALS AND METHODS

### Experimental design

The objective of this study was to characterize the spatiotemporal protease activity landscape involved in inflammatory lung cancer and identify key contributors to cancer progression to support the development of an interventional therapy. Protease substrates were designed and screened for recombinant interleukin- and cancer-associated protease cleavage to nominate specifically and efficiently cleaved sequences. We investigated changes in protease activity with IL-1b blockade in KPS mice with substrates translated into in vivo ABNs. We explored protease candidates from differentially cleaved ABN hits, e.g., caspase-1, in situ in KPS lung tissue. Given elevated caspase-1 expression and activity in early KPS tumors, we assessed whether early systemic inhibition of caspase-1 and IL-1b could reduce tumor development in vivo. Timelines for in vivo experiments were prospectively determined. All experiments performed had at least three biological replicates. Detailed methods for experiments are described below and in each figure legend as appropriate.

All animal studies and procedures were approved by the Committee for Animal Care at Massachusetts Institute of Technology (MIT) (#2301000462 and #2212000452) and were conducted in compliance with institutional and national policies. All animal studies were performed in compliance with Animal Research: Reporting In Vivo Experiments (ARRIVE) guidelines.

Schematic images were generated using Adobe Illustrator and Microsoft PowerPoint.

### FRET assay

FRET probes were ordered from CPC Scientific (Sunnyvale, CA) and designed to have the quencher 2,4-dinitrophenyl-lysine (DNP) and fluorophore 7-methoxycoumarin-4-acetic acid (MCA) or 6-carboxyfluorescein (FAM) on either side of the cleavable peptide sequences. FRET probes (50 μM) were mixed with recombinant proteins (10 nM) or lung homogenate (total protein concentration: 1 mg/ml) to initiate the cleavage reaction, and the fluorescence signal was read out periodically with a Tecan plate reader for over an hour. Recombinant proteases screened included caspase-1 (ALX-201-056-U025, Enzo), caspase-3 (707-C3, R&D Systems), caspase-7 (823-C7, R&D Systems), caspase-8 (ALX-201-163-C020, Enzo), cathepsin B (965-CY, R&D Systems), CTSG (BML-SE283-0100, Enzo), chymase-1 (4099-SE, R&D Systems), chymotrypsin (6907-SE, R&D Systems), granzyme B (1865-SE, R&D Systems), meprin A (4007-ZN, R&D Systems), meprin B (3300-ZN, R&D Systems), and ELANE (4517-SE, R&D Systems). For FRET with lung homogenate samples, chemical inhibitors were added to validate protease activity-enabled fluorescence. A 100x dilution of Halt Protease Inhibitor Cocktail (78429, Thermo Fisher Scientific) and 1 mM marimastat (HY-12169, MedChemExpress) were used together for pan-protease inhibition. A 1 mM VRT-043198 (HY-112226, MedChemExpress) solution was used for caspase-1–specific inhibition. Cleavage efficiency and specificity were quantified as standardized scores across substrates and across proteases, respectively, as previously described (*22*).

### KPS murine model

To develop the KPS LUAD murine model, we generated *Kras*^LSL-G12D^;*Trp53*^fl/fl^ C57BL/6 (KP) mice in which the activation of an oncogenic allele of *Kras* is sufficient to initiate the tumorigenesis process, and additional deletion of *Trp53* substantially enhances tumor progression (*44*). LUAD lung tumors were initiated in KP mice that were 8 to 12 weeks old by intratracheal administration of 10,000 plaque-forming units of lentivirus expressing Cre recombinase and a tumor-specific neoantigen SIINFEKL fused to the fluorophore mScarlet (Lenti-SIIN) (*24*). The mice receiving Lenti-SIIN were then randomly grouped to minimize the variation in viral delivery. Healthy control cohorts consisted of age- and sex-matched mice. Tumor growth was allowed in KPS mice until the treatment or detection was performed. During selection of KPS mice for treatment groups, investigators were blinded to all characteristics but age, sex, and genotype.

### In vivo treatment studies

To evaluate IL-1b blockade in KPS mice, all tumor-bearing mice received one of the following treatment regimens at the end of 5 weeks post–LUAD initiation: IgG control, anti-IL-1b, anti-PD-1, and anti-IL-1b with anti-PD-1. The antibodies, anti-Armenian hamster IgG (BE0091, InVivoMAb), anti-mouse/rat IL-1b (BE0246, InVivoMAb), and anti-mouse PD-1 (BP0273, InVivoMAb), were administered three times a week intraperitoneally at 10 mg/kg. The treatment continued for 9 weeks, with longitudinal monitoring of tumor growth by micro-CT every 2 or 3 weeks and ABN administration after 3 weeks of treatment.

To test caspase-1 inhibition in vivo, KPS mice were treated 4 weeks post–lentiviral induction. The treatment groups were IgG with drug vehicle, anti-IL-1b with drug vehicle, IgG with belnacasan, and anti-IL-1b with belnacasan. Mice were dosed with antibody intraperitoneally three times a week at 10 mg/kg. Belnacasan (HY-13205, MedChemExpress) was solubilized in the vehicle (20% Kolliphor in water) at a concentration of 200 mg/ml. Mice were administered belnacasan (100 mg/kg) or vehicle control every day via oral gavage, starting on the same day as the antibody treatment. The treatment continued for 7 weeks, with longitudinal monitoring of tumor growth by micro-CT every 2 or 3 weeks.

### Intratracheal ABN administration

Mass-encoded ABNs were synthesized and characterized by CPC Scientific (Sunnyvale, CA), consisting of glutamate–fibrinopeptide B (GluFib; EGVNDNEEGFFSAR) reporters with different distributions of stable isotopes linked to an eight-arm 40-kDa PEG via unique amino acid sequence linkers. For intratracheal instillation, a 20-plexed mass-encoded ABN cocktail (20 μM substrate) in phosphate buffer [0.28 M mannitol, 5 mM sodium phosphate monobasic, and 15 mM sodium phosphate dibasic (pH 7.2 to 7.4)] was administered by solution injection following intratracheal intubation with a 22-gauge flexible plastic catheter (Exel International, Redondo Beach, CA). One hundred microliters of air was then pushed into mouse lungs via a 1-ml syringe to ensure a full delivery of ABNs into the lungs. A subcutaneous injection of 200 μl of sterile phosphate-buffered saline (PBS) was administered in favor of urine production. Bladders were voided at 60 min after nanosensor administration, and urines produced from 60 to 120 min after administration were collected. All urine samples were immediately frozen at −80°C until analysis by a quadruple liquid chromatography–tandem mass spectrometry in the Biopolymers and Proteomic Core at the Koch Institute for Integrative Cancer Research.

### Lung tissue collection and preparation

For frozen tissue sections, mouse lungs were collected and embedded in Optimal Cutting Temperature (OCT) compound blocks. OCT blocks were frozen over dry ice and stored at −80°C for cryosectioning. For histology, collected mouse lungs were fixed in 4% paraformaldehyde before paraffin-embedding for sectioning and hematoxylin and eosin (H&E) staining. BALF samples were collected by intratracheally instilling 1 ml of PBS and suctioning the contents. They were then spun down and removed of cell pellet.

### Immunofluorescence and AZP staining of frozen tissue

The cleavable peptide sequence was ordered from CPC Scientific (Sunnyvale, CA), flanked by a negatively charged E9 domain and a positively charged R9 domain, with the Cy5 fluorophore conjugated to the end of the positive domain. A polyR peptide consisting of the R9 domain with a Cy7 fluorophore was used as a positive control for electrostatic binding. Staining with AZPs was performed as previously described (*18*). Briefly, frozen tissue sections were prepared by washing with PBS and fixing with ice-cold acetone. They were then blocked with 1% bovine serum albumin at room temperature before incubating with the AZP probes and primary antibodies, if applicable, for 4 hours at 37°C. For studies with only antibody staining, tissues were blocked in the same manner and incubated with antibodies overnight at 4°C. The tissues were then washed with PBS and incubated with secondary antibodies diluted in PBS for 30 min at room temperature. Primary antibodies used include caspase-1 (22915-1-AP, Thermo Fisher Scientific), CTSG (PA5-99402, Thermo Fisher Scientific), ELANE (PA5-115648, Thermo Fisher Scientific), CD3 (R&D Systems, MAB4841), CD31 (R&D Systems, AF3628), CD68 (Bio-Rad, MCA1957), E-cadherin (R&D Systems, AF748), and Ly-6G (Thermo Fisher, MA1-82826). For studies including tissue protease inhibition, inhibitors were coincubated with the blocking solution and with the AZP probes. A 100x dilution of Halt Protease Inhibitor Cocktail (78429, Thermo Fisher Scientific), 1 mM marimastat (HY-12169, MedChemExpress), and 1 mM 4-(2-aminoethyl)benzenesulfonyl fluoride hydrochloride (AEBSF) (A8456, Millipore Sigma) were used together for pan-protease inhibition. A 1 mM belnacasan (HY-13205, MedChemExpress) solution was used for caspase-1–specific inhibition. For quantification, cells with fluorescence intensity signals above a predetermined threshold were detected as antibody marker+ using QuPath 0.5.1 (*45*). AZP signals were quantified by normalizing the AZP fluorophore signal with the polyR Cy7 signal for each cell.

### Immunohistochemical staining of fixed tissue

Slides with formalin-fixed, paraffin-embedded tissue sections were deparaffinized with xylene, rehydrated, and antigen retrieved in at 97°C for 20 min using citrate heat-induced epitope retrieval buffer (pH 6). They were then blocked for 30 min, followed by incubation with primary antibody for 1 hour, horseradish peroxidase (HRP) for 30 min, and 3,3′-diaminobenzidine (DAB) substrate for 5 min. Primary antibodies for IL-1b (218753, Bio-X-Cell), F4/80 (70076, Cell Signaling Technologies), ASC/PYCARD (PA5-50915, Thermo Fisher), and caspase-1 (MA5-16215, Thermo Fisher Scientific) were diluted with tris-buffered saline with Tween 20, and HRP was used for secondary detection.

### Human LUAD sample processing

Sections from fresh frozen human LUAD samples and NAT were acquired from the Massachusetts General Hospital Lung Tumor Bank. Deidentified BALF samples from patients with LUAD and from healthy donors were purchased from Bay Biosciences LLC (Brookline, MA). Human BALF samples were assessed for human caspase-1 expression with a solid-phase sandwich ELISA kit (DCA100, R&D Systems), as described in the kit protocol. Caspase-1 activity in BALF was assessed with a bioluminescence-based assay, as described in the kit protocol (G9951, Promega). Cells with positive staining for DAB were detected and quantified with QuPath 0.5.1 (*45*).

### Statistics

Statistical analyses were performed using GraphPad Prism v10. Data are generally presented as means ± SDs in the figures. One-way analysis of variance (ANOVA) with Tukey’s multiple comparison test was used for statistical testing unless noted otherwise. Statistical significance is indicated as *P < 0.05, **P < 0.01, ***P < 0.005, and ****P < 0.001. 

#### Note added in proof:

While this paper was in press, Cell published a study by Pandya *et al.* (*46*) wherein the researchers used machine learning to define a 14-protein plasma signature that, when applied to CANTOS patients, identified individuals who seemed to benefit more from anti-IL-1β therapy.
