# Supplementary material for: Multimodal profiling of pro-inflammatory protease activity identifies caspase-1 as a target for lung cancer interception
Source: Sci Adv. 2026 Aug 14;12(33):eadz4263. doi: 10.1126/sciadv.adz4263 (PMC13475574; doi:10.1126/sciadv.adz4263)
Supplement: Supplementary file 1 [file sciadv.adz4263_sm.pdf]

Science Advances
AAAS

Supplementary Materials for

Multimodal profiling of pro-inflammatory protease activity identifies caspase-1 as a target for lung cancer interception

Cathy S. Wang *et al.*

Corresponding author: Sangeeta N. Bhatia, sbhatia@mit.edu

Sci. Adv. **12**, eadz4263 (2026)
DOI: 10.1126/sciadv.adz4263

The PDF file includes:

Figs. S1 to S15
Tables S1 to S3
Legend for data S1

Other Supplementary Material for this manuscript includes the following:

Data S1

Supplementary Figures

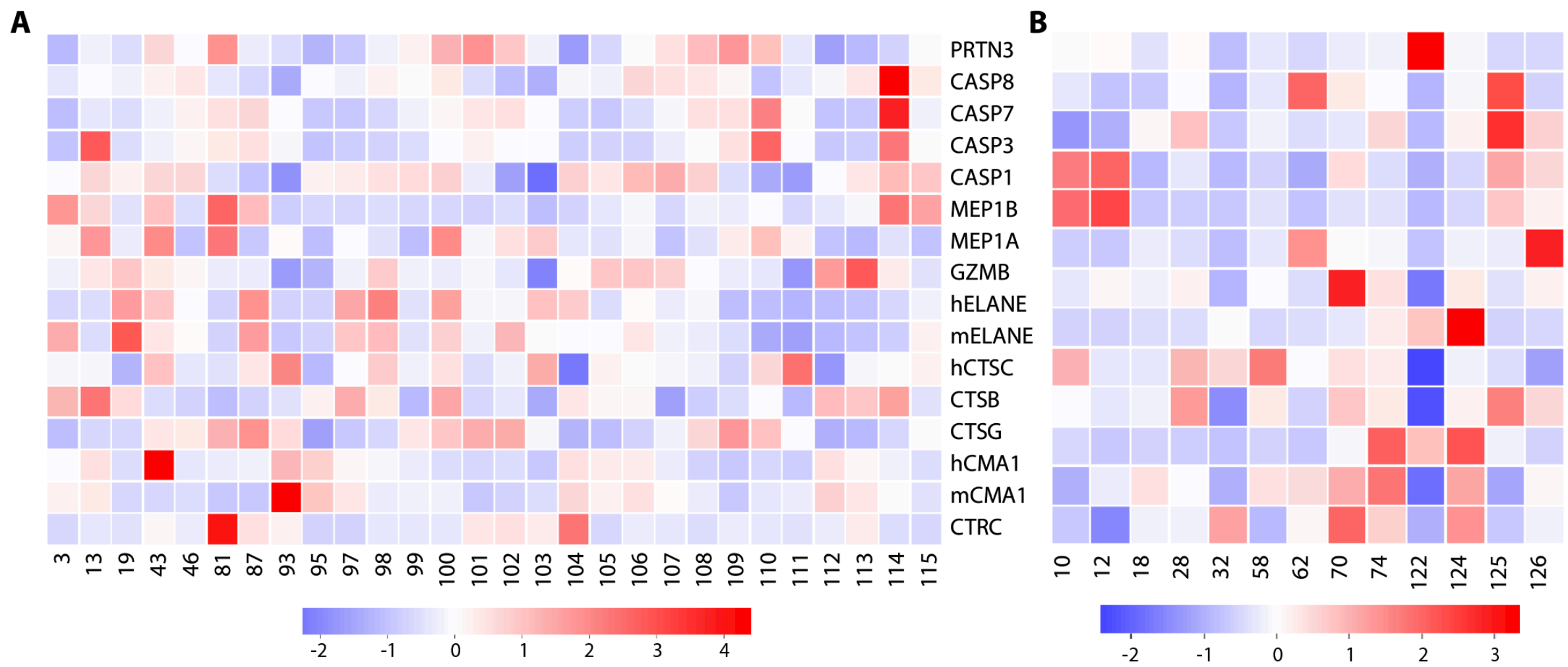

Figure S1. Peptide substrate cleavage screening. Heatmaps depicting z-scored cleavage fold change of FRET substrates (columns, n=3 technical replicates) in replicate experiments by (**A**) interleukin- or (**B**) a combination of interleukin- and cancer-associated proteases (rows). Proteases are mouse recombinant proteins unless noted otherwise. PRTN3: proteinase-3, CASP1/3/7/8: caspase-1/3/7/8, MEP1A/B: meprin A subunit alpha/beta; GZMB: granzyme B, h/mELANE: human/mouse neutrophil elastase, CTSB/C/G: cathepsin B/C/G; h/mCMA1: human/mouse chymase, CTRC: chymotrypsin C, MMP7: matrix metalloprotease-7, FAP: fibroblast activation protein.

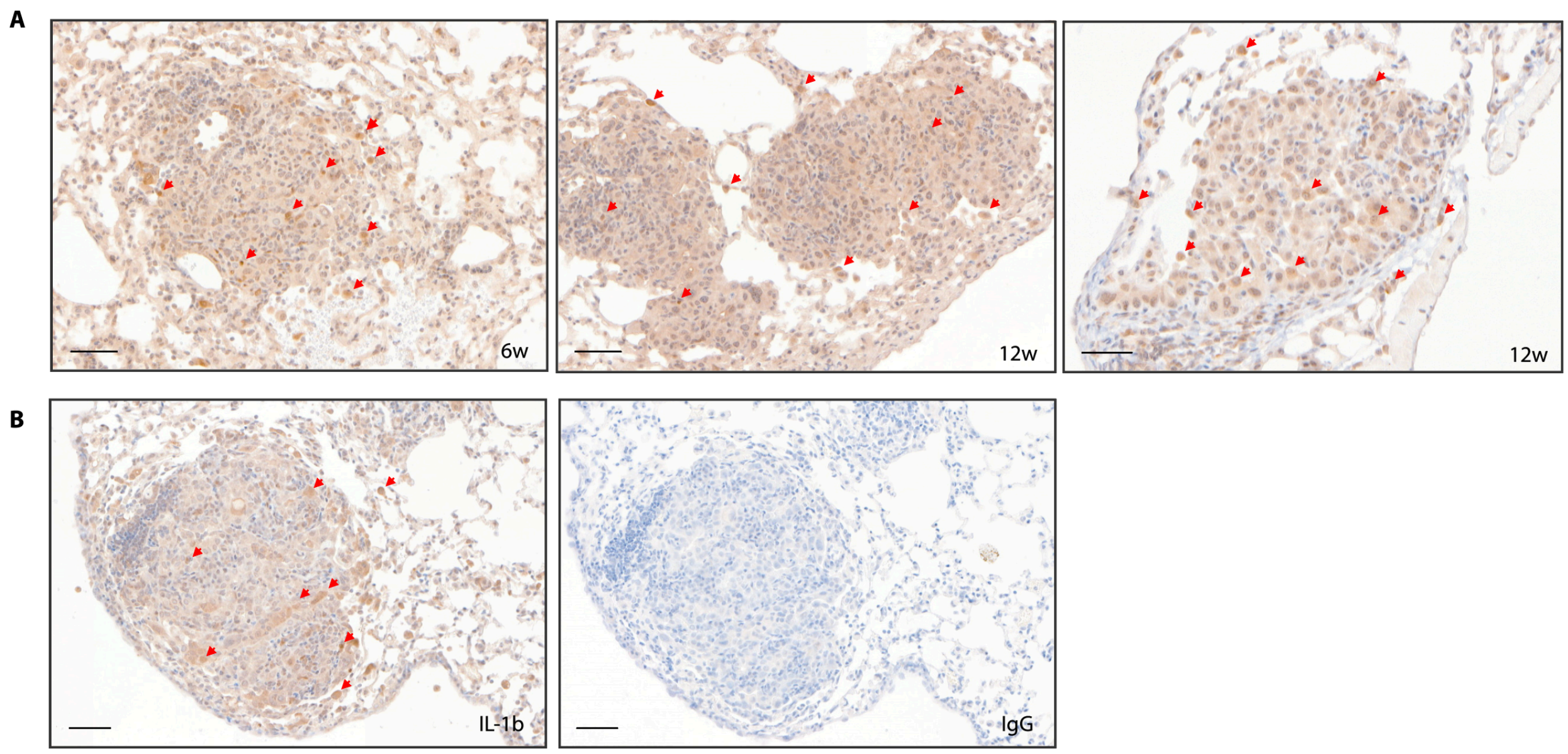

Figure S2. Immunohistochemical staining of IL-1b. (**A**) Dark brown stained cells (examples shown by red arrows) show IL-1b expression in 6- and 12-week KPS lung tissue. Scale bar 50um. (**B**) Comparison of IL-1b staining (left) and IgG control staining (right) in 6-week KPS lung tissue from an independent experiment. Scale bar 50um.

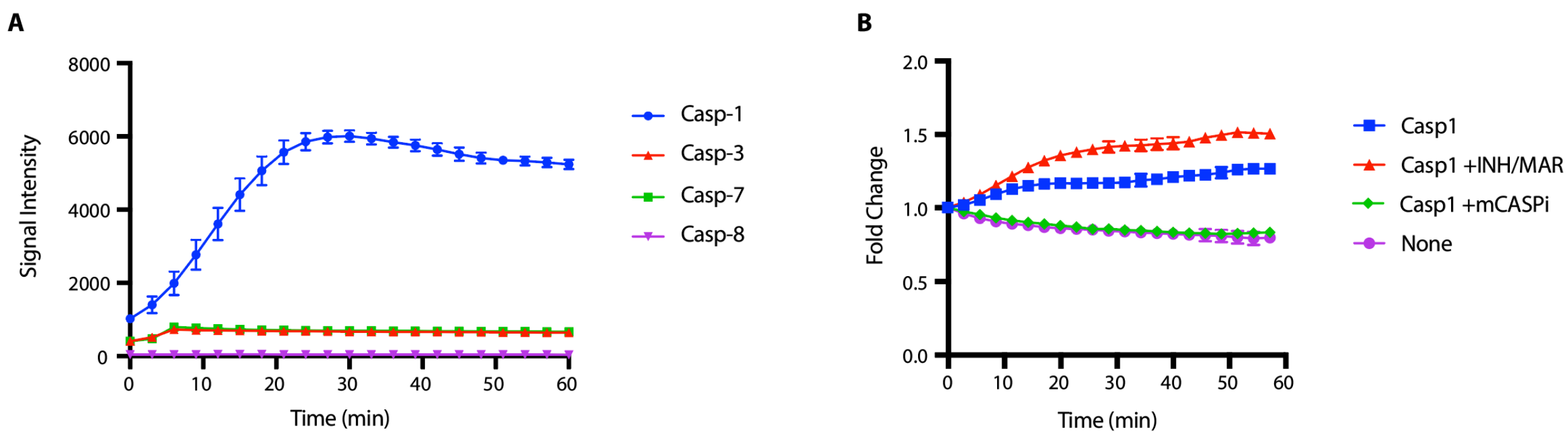

Figure S3. Caspase-specific cleavage of A10 FRET probe. (**A**) A10 was incubated with recombinant caspase-1 (blue), -3 (red), -7 (green), -8 (purple), where fluorescence signal intensity indicates FRET probe cleavage. n=3 technical replicates. (**B**) Fold change in fluorescence signal indicates cleavage of A10 incubated by recombinant caspase-1 (blue), caspase-1 with broad-spectrum inhibitor (INH) and marimastat (MAR) (red), or caspase-1 with metabolized caspase-1 inhibitor (mCASPi, green). A10 alone (None, purple) was used as a negative control. n=3 technical replicates.

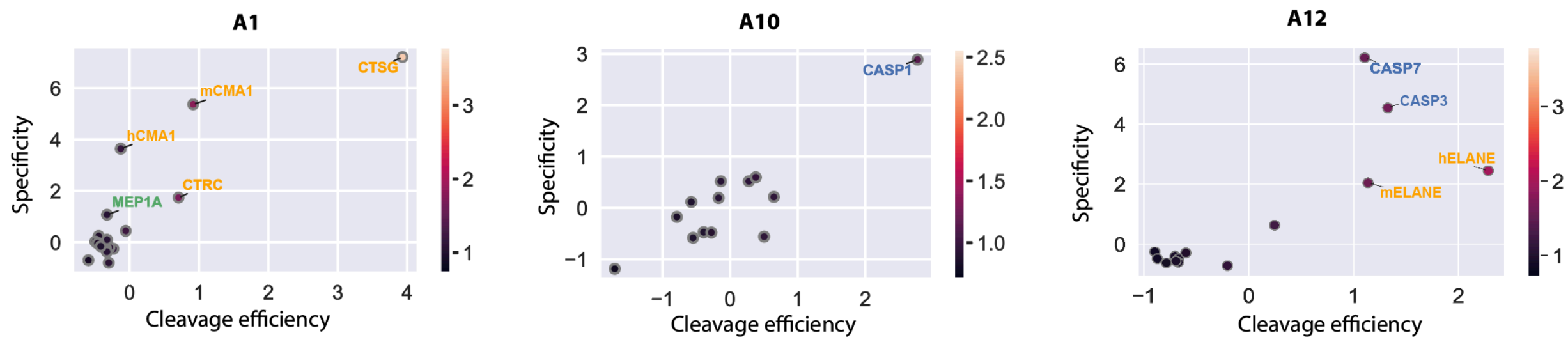

Figure S4. Cleavage efficiency versus specificity plots for substrates of interest. Standardized cleavage fold changes (n=3 technical replicates) of different proteases are compared for A1, A10, and A12, aggregated across three independent experiments. A1 is highly cleaved by cathepsin G (CTSG), A10 by caspase-1 (CASP1), and A12 by neutrophil elastase (ELANE), and caspase-3/7 (CASP3/7). h-: human recombinant protein, m-: mouse recombinant protein. Text color indicates protease class, where green: matrix metalloprotease, yellow: serine, blue: cytosine.

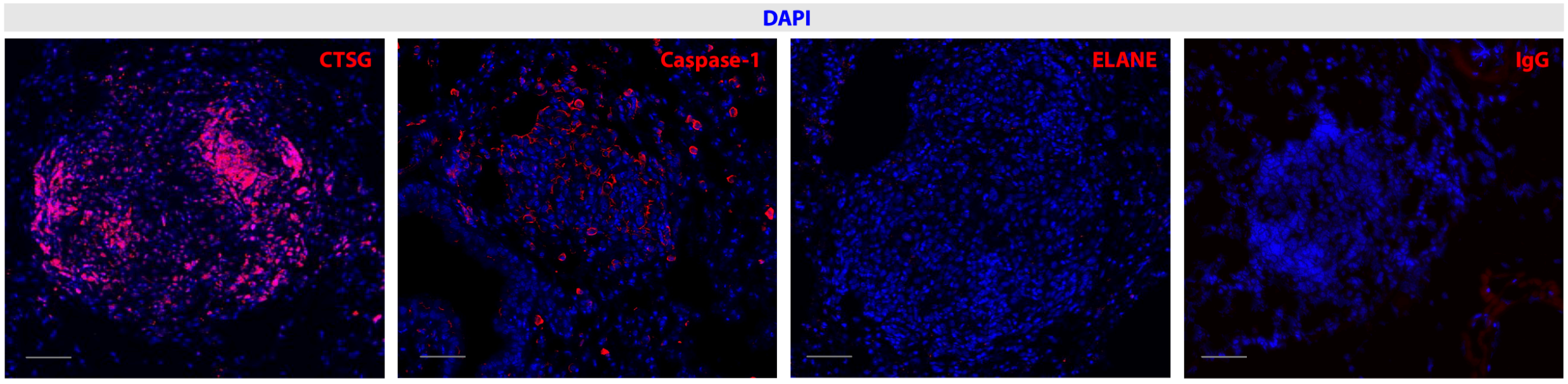

Figure S5. Immunofluorescence staining of proteases. Cathepsin G (CTSG), caspase-1, neutrophil elastase (ELANE), and IgG (red) are each co-stained with a nuclear dye (DAPI, blue) in 12-week KPS tumors. Scale bar 50um.

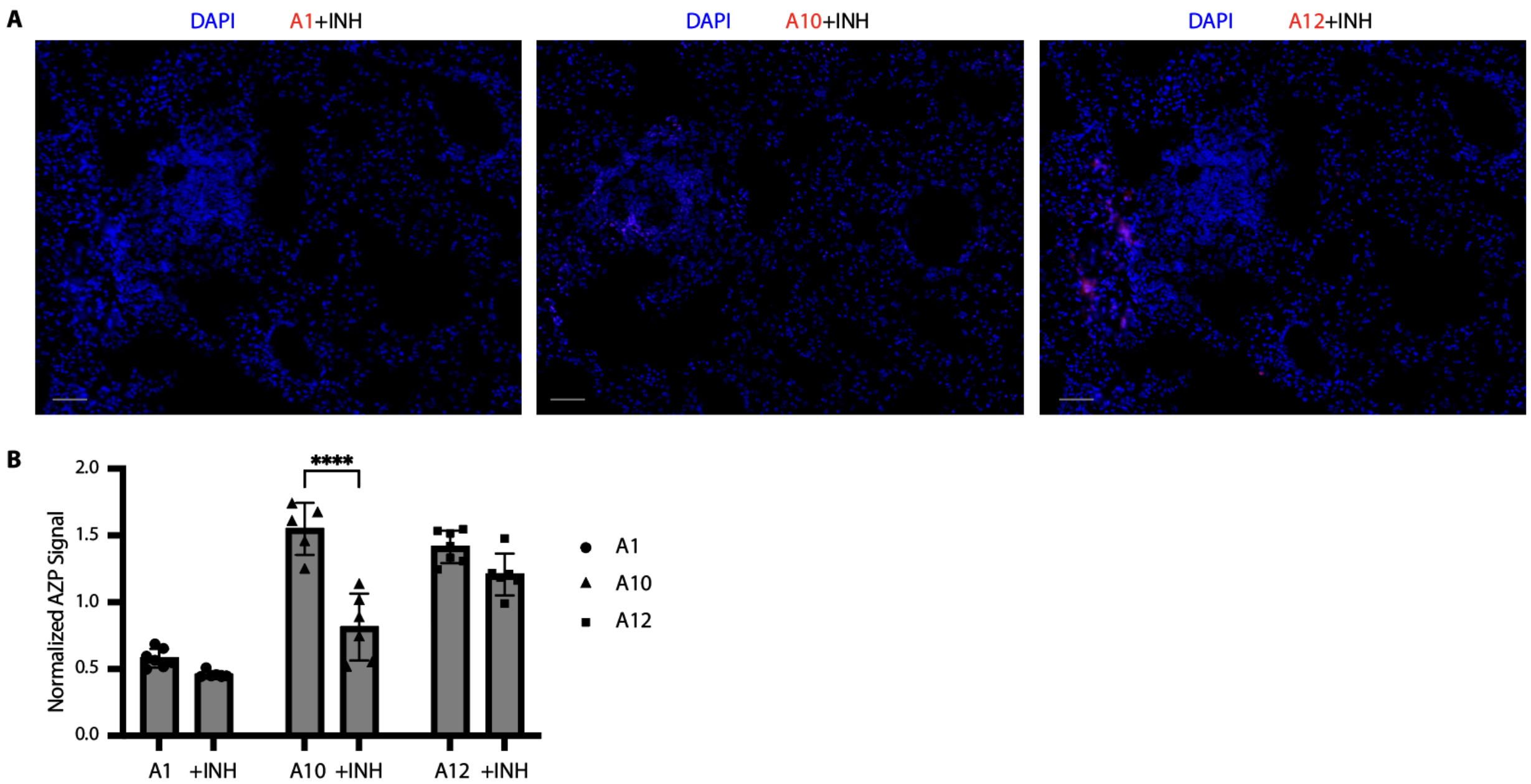

Figure S6. Co-staining of AZPs with inhibitors on KPS tissue. (**A**) A1, A10, and A12 AZPs (red) are each incubated with broad-spectrum protease inhibitor (INH) on 6-week KPS tissue to show cleavage-specific staining. Scale bar 50um. (**B**) Normalized AZP signal with and without INH co-incubation (n=5-7 tumor regions) was quantified. Two-way ANOVA with Sidak's multiple comparison test was performed ($p < 0.05$).

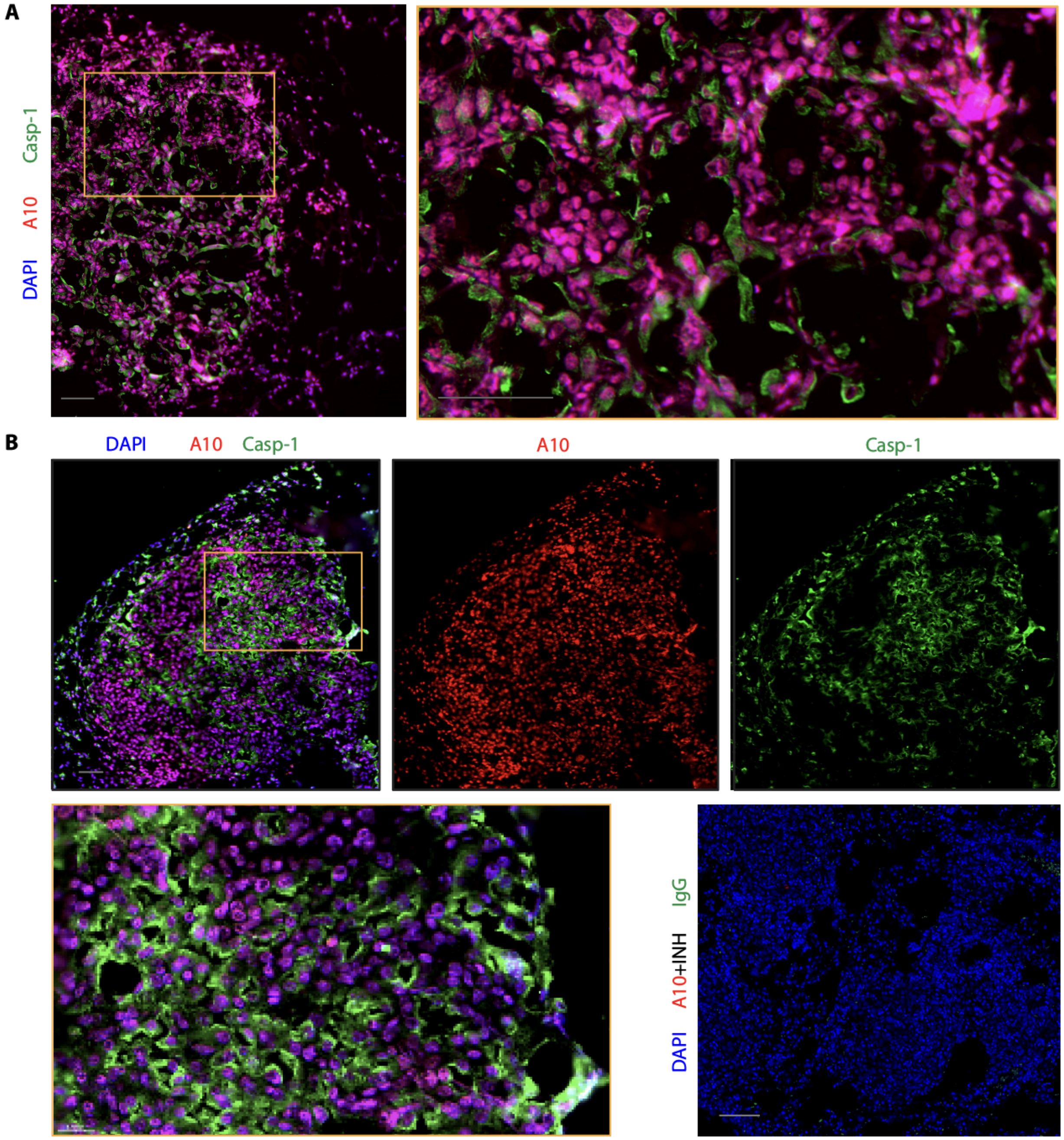

Figure S7. Caspase-1 protein expression and activity staining. Overlayed images of nuclear dye (DAPI, blue), AZP (A10, red), and caspase-1 (green) in (**A**) 6-week and (**B**) 12-week KPS tumors. Co-incubation with broad-spectrum inhibitor (INH) and IgG (green) show nonspecific, control staining. Scale bar 50um unless otherwise indicated.

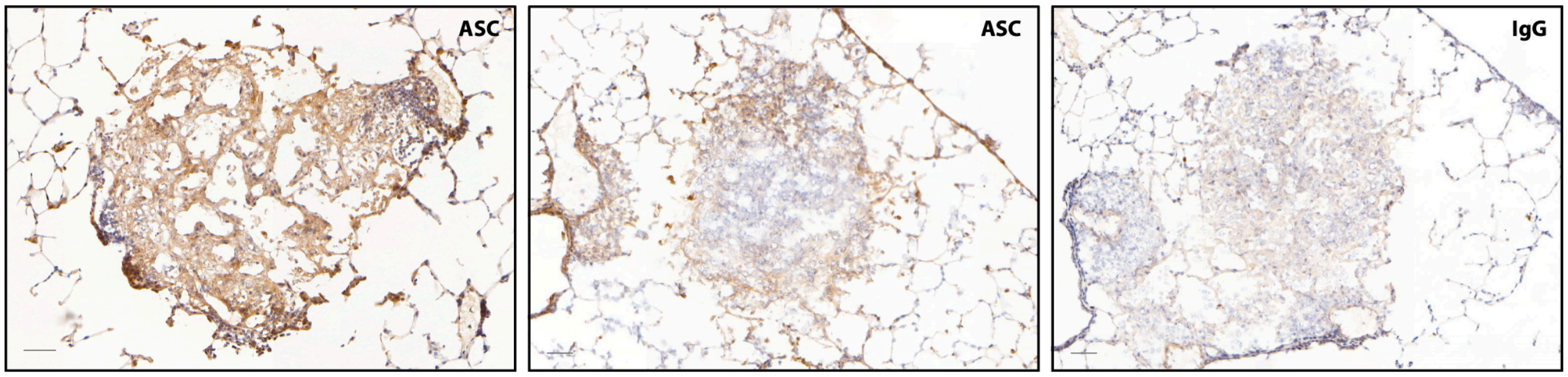

Figure S8. Immunohistochemical staining of ASC. Dark brown stained cells show ASC expression, indicative of inflammasome formation, (left, middle) in lung tissue from two 6-week KPS mice. IgG staining (right) is shown as control. Scale bar 50um.

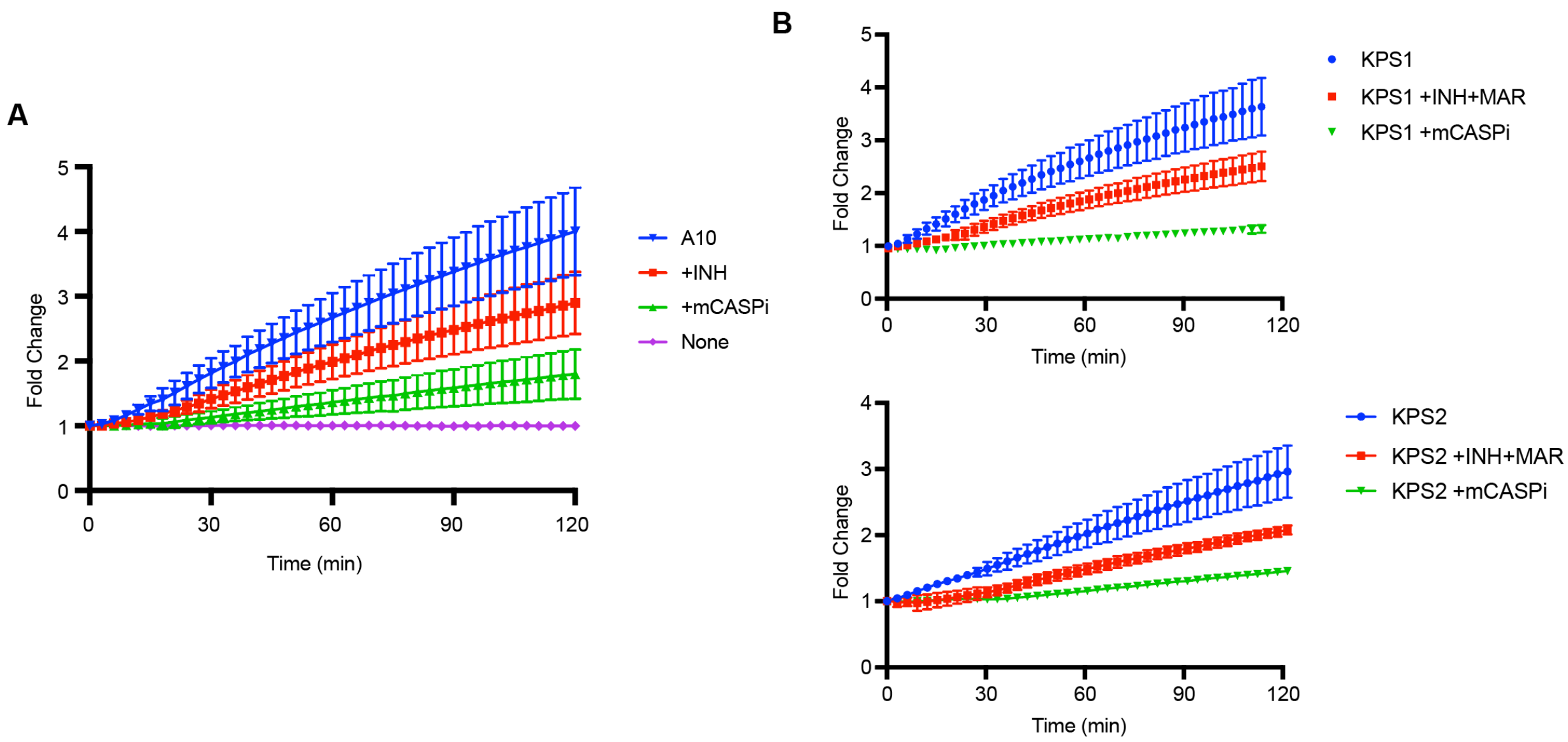

Figure S9. FRET cleavage of A10 with lung homogenates from 12-week-old KPS mice. (**A**) Fold change in fluorescence signal indicating cleavage of A10 FRET probe by lung homogenates (n=3 mice) with and without broad-spectrum inhibitor (INH) and marimastat (MAR) or metabolized caspase-1 inhibitor (mCASPi). (**B**) Examples of probe cleavage (n=2-3 technical replicates) in individual mice.

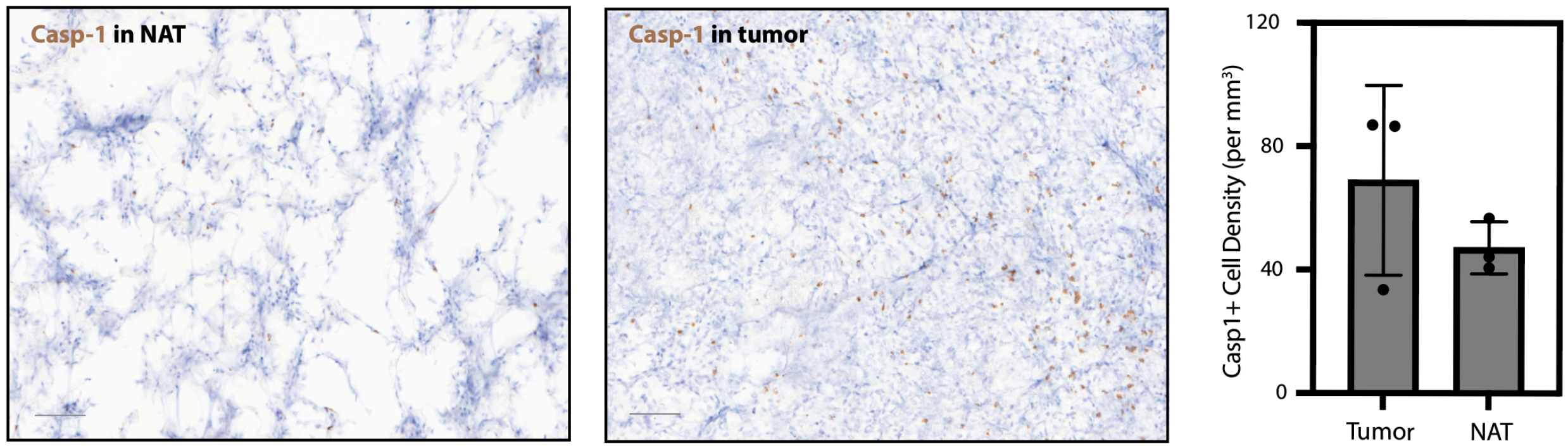

Figure S10. Immunohistochemical staining of caspase-1 in human LUAD biopsy samples. Caspase-1 (brown) expression was compared between normal tissue adjacent to tumor (NAT) and tumor regions. Scale bar 100um. Positive cell density (per mm^2) for caspase-1 expression was quantified (n=3 donor samples).

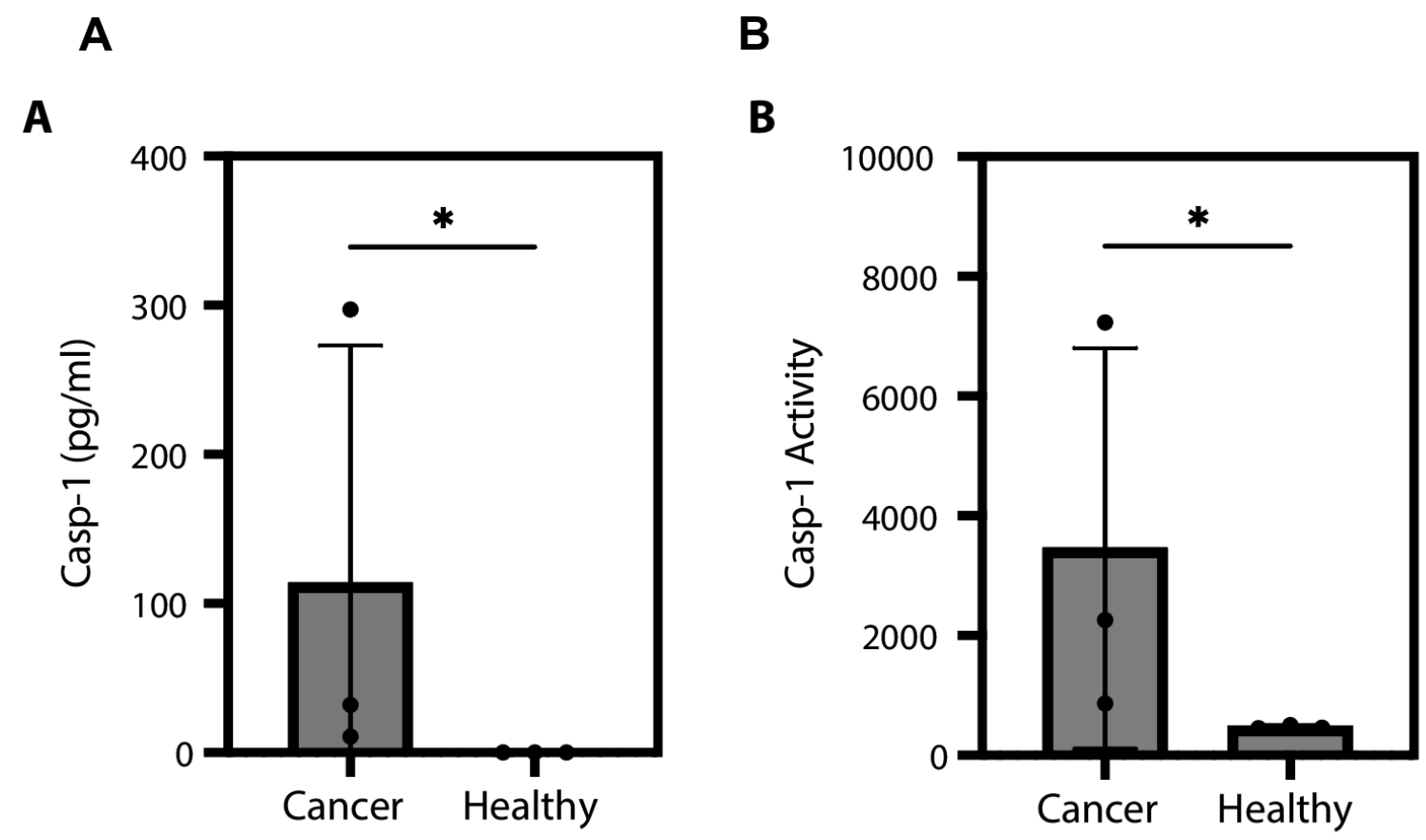

Figure S11. Characterization of caspase-1 expression and activity in human bronchioalveolar lavage fluid (BALF). (A) Caspase-1 expression in BALF from LUAD patients (n=3) and from healthy smokers (n=3) was quantified by ELISA. Mann-Whitney U test was performed ($p = 0.05$). (B) Caspase-1 activity in BALF (n=3 donor samples per group) was assessed with a commercial luminescence kit. Mann-Whitney U test was performed ($p = 0.05$).

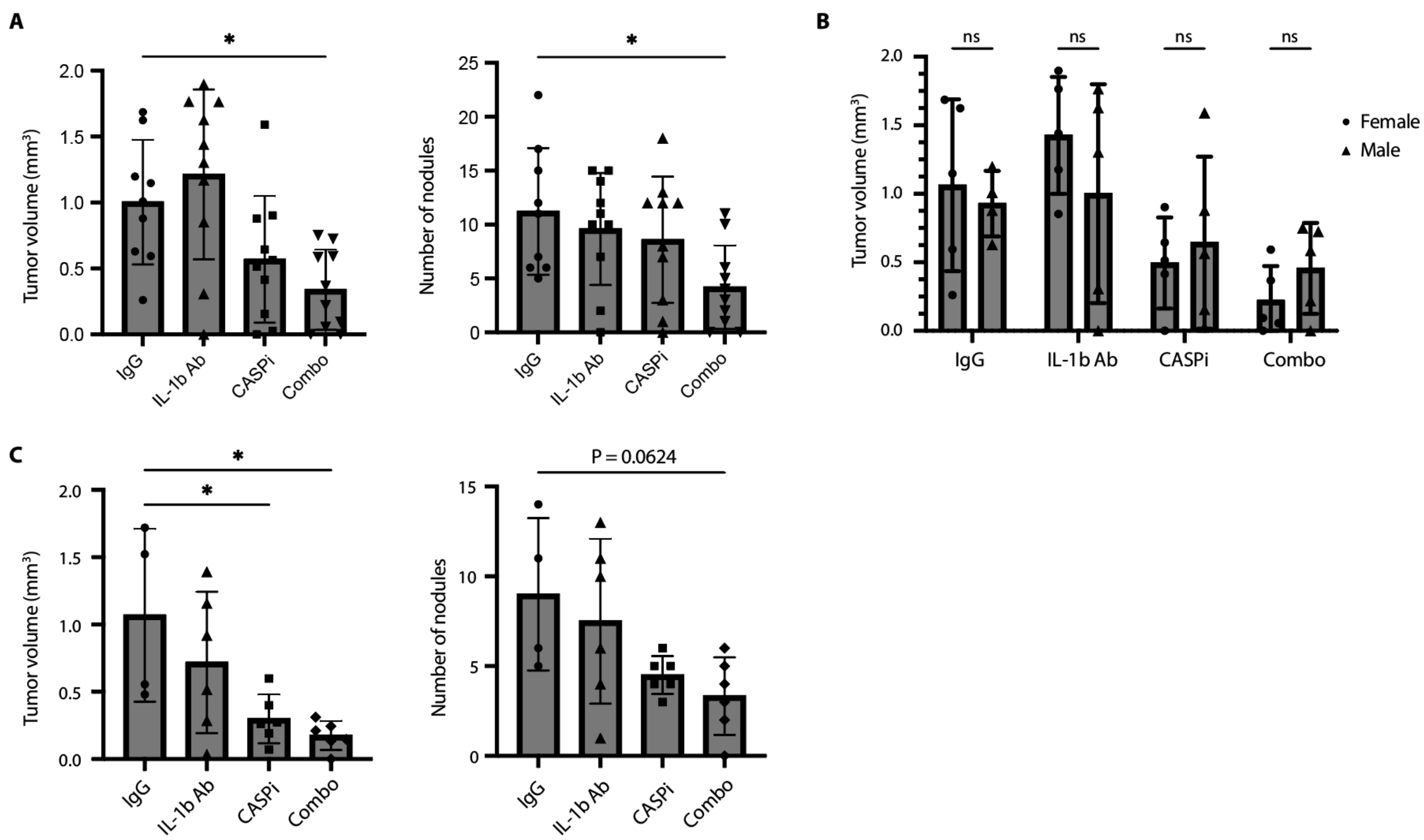

Figure S12. Treatment of independent KPS cohorts. (A) Tumor volume quantification and nodule count in first cohort of KPS mice (n=9-10 mice per group) after seven weeks of treatment with IgG, IL-1b antibody, belnacasan (CASPi), and IL-1b antibody and belnacasan (combo). One-way ANOVA with Tukey's multiple comparison test was performed ($p < 0.05$). (B) Separation of first cohort treatment groups by sex shows no significant difference in treatment effect. Two-way ANOVA with Fisher's LSD test was performed ($p < 0.05$). (C) Tumor volume quantification and nodule count of second cohort of KPS mice (n=4-6 mice per group) after seven weeks of treatment. One-way ANOVA with Tukey's multiple comparison test was performed ($p < 0.05$).

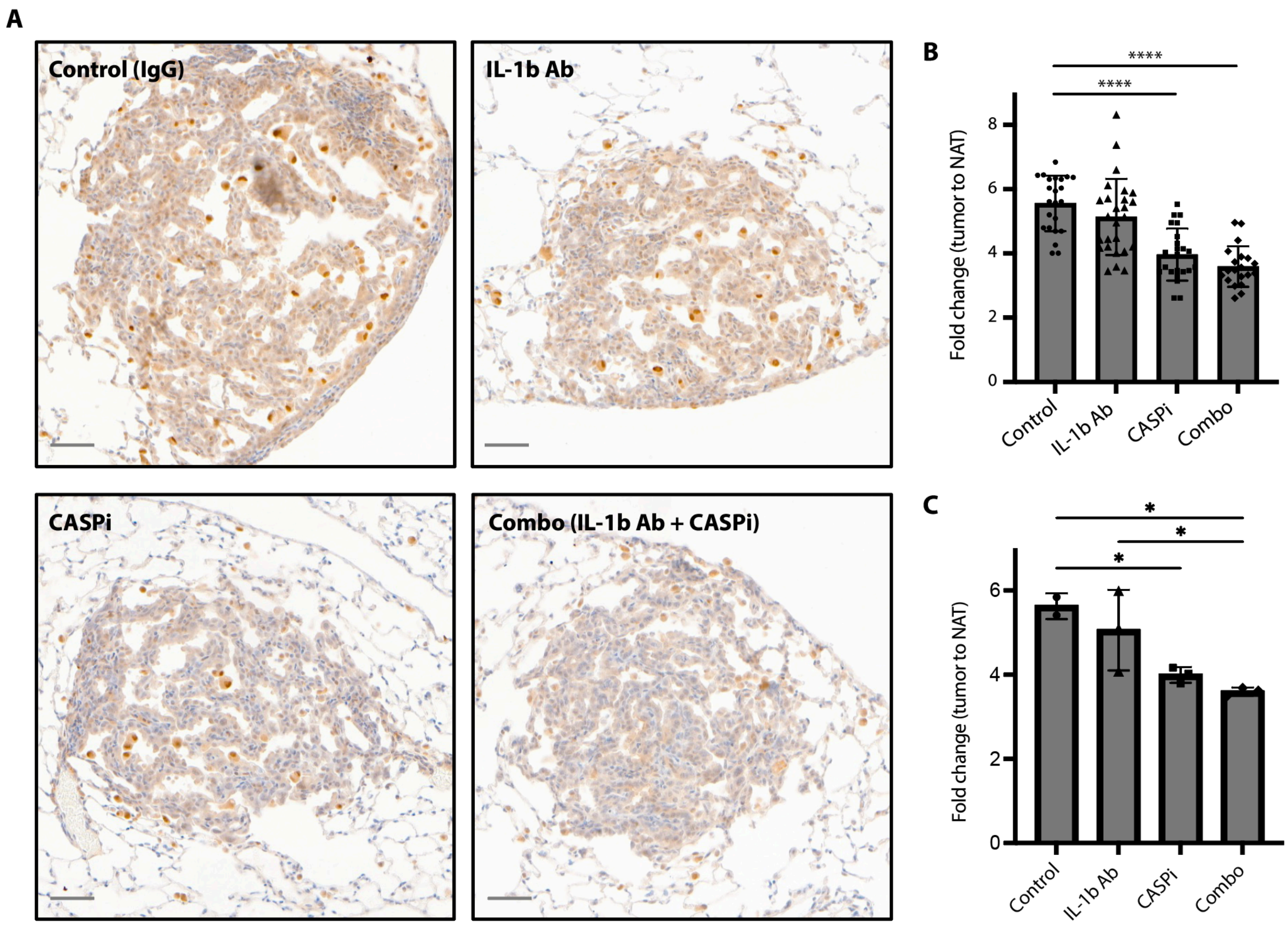

Figure S13. Immunohistochemical staining of active IL-1b in treated KPS tumors. (A) IL-1b (dark brown) expression was compared across treatment groups. Scale bar 50um. (B) Fold change of mean signal intensity in tumor over NAT was quantified showing (B) n=20-26 regions and (C) average fold change from n=2-3 mice per treatment group. One-way ANOVA with Dunnett's multiple comparison test was performed ($p < 0.05$).

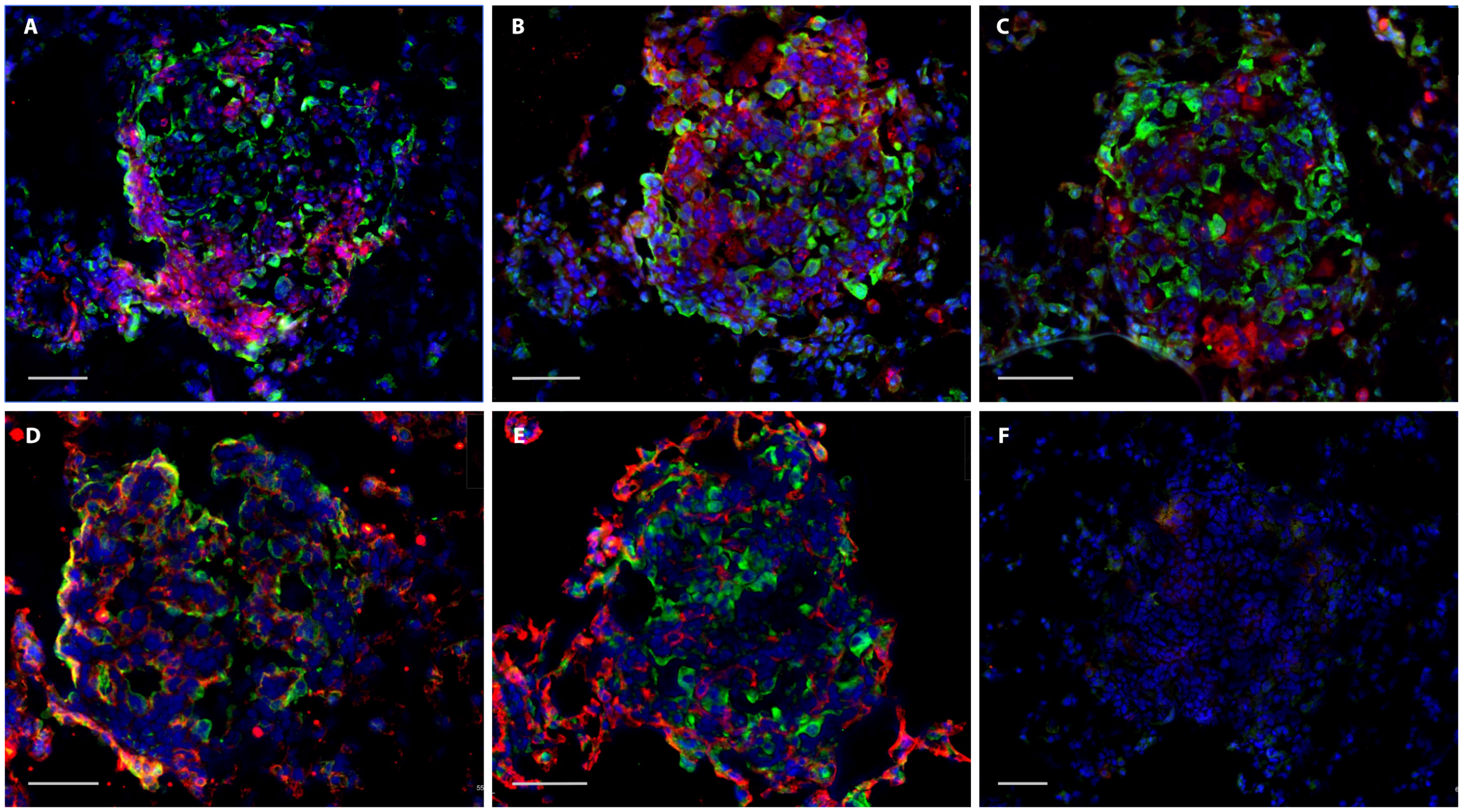

Figure S14. **Immunofluorescence co-staining of caspase-1 with cell markers in early-stage KPS tumors.** DAPI nuclear stain (blue) and caspase-1 (green) are co-stained with separate cell markers (red) for (A) T cells, CD3, (B) neutrophils, Ly-6G, (C) macrophages, CD68, (D) epithelial cells, E-cadherin, (E) endothelial cells, CD31, and (F) control IgG, in subsequent frozen sections of 7-week KPS tumor tissue. Scale bar 50um.

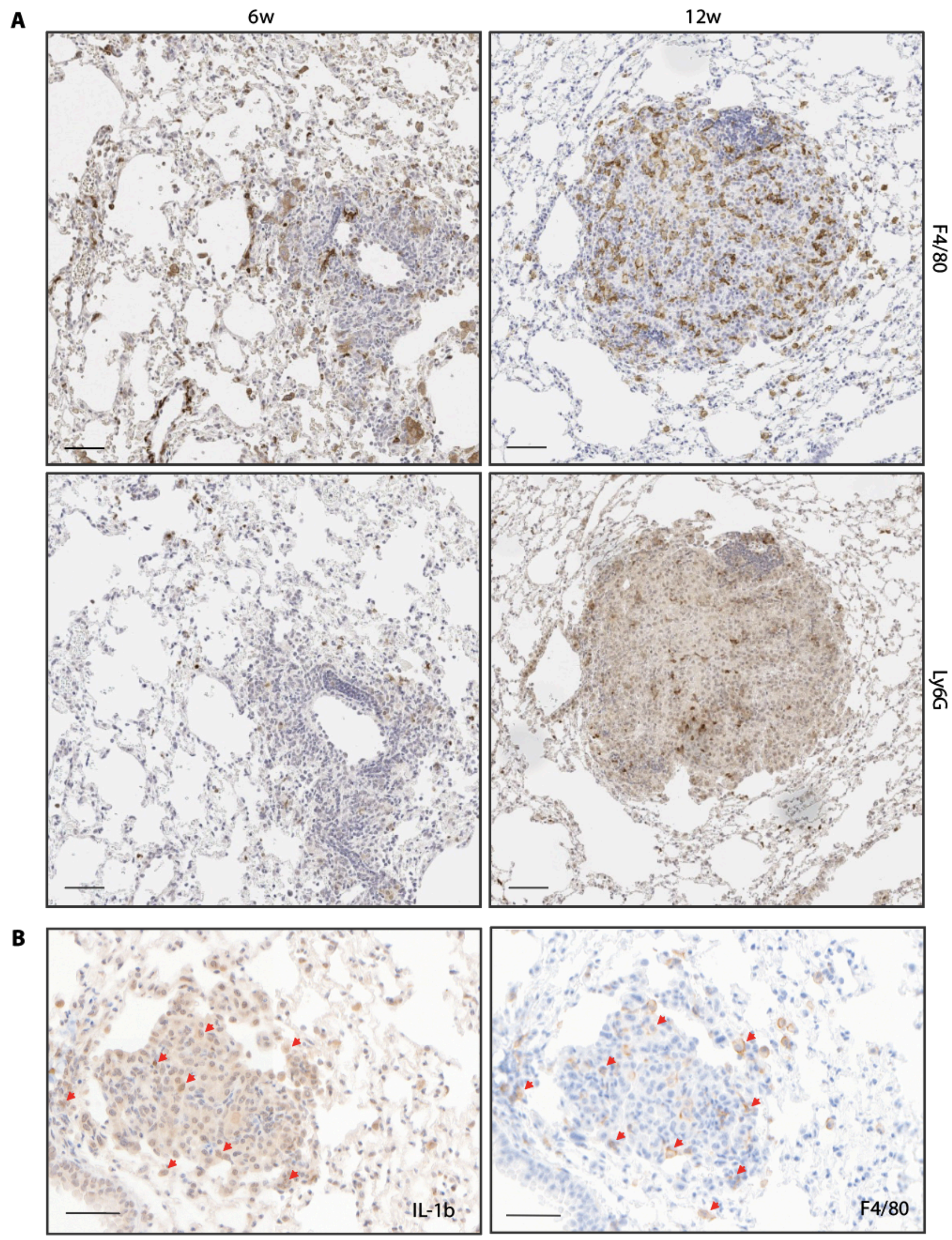

Figure S15. Immunohistochemical staining of immune cell populations. (A) Macrophage (F4/80) or neutrophil (Ly6G) infiltration (dark brown) was compared in untreated 6- and 12-week KPS lung tissue. Scale bar 50um. (B) IL-1b and macrophage (F4/80) staining in 12-week KPS lung tissue. Scale bar 50um.

Table S1. Nominated peptide sequences designed to be responsive to interleukin-processing proteases. Successfully synthesized substrates were used for in vitro recombinant protease FRET screens. Uppercase: L-isomer amino acids; lowercase: D-isomer amino acids; AIBA: α-Aminoisobutyric acid.

Substrate label	Sequence (C-terminus = CONH2)
1	GWEHDGG
2	GWEHDKGG
3	YVADAP
4	YVHDAPGG
5	YVADAPV
6	GYVADAPVGG
7	GYVHDAPVGG
8	GLVCDVGG
9	GNLLVCDVPG
10	VDQVDGW
11	DEVDAP
12	VDEVDGW
13	VDQMDGW
14	EPLFAAR
15	GPLFAGRGG
16	GIEPDGG
17	GIEPDKGG
18	GIETDGG
19	IEPDVSQV
20	GGIVRAGG
21	GFLG
22	GGFLGG
23	GGFGFVGG
24	GGFAFGG
25	EPFWEDQ
26	GEPFWEDQG
27	GIEPKSDPMPEQ
28	TPFSGQ
29	GTPFSGQGG
30	GAAPLGG
31	VVLDSEVL
32	GAYVHGG
33	GEAYVHGG
34	VADvRDRQ
35	GVADvRDRQG
36	VANvAERQ
37	GVANvAERQG
38	VADCADQ
39	GVADCADQG
40	GYY[AIBA]NEPGG
41	VADCRDRQ
42	VARCADYQ
43	VADvADYQ
44	MLDAMGSL
45	VRRVVQYQD
46	GDNLLVC GG
47	APEEIMRRQ
48	HPVPVYAFSPQ
49	QPMAVVQSVPQ
50	GAAPVGG
51	GAVVASELR
52	TLLVSGNA
53	RQRVNWNW
54	GFIFEEEPIFF G
55	GEEEPIFGG
56	GFIFEEEPILCG
57	GEEEPILGG
58	GVDVADGG
59	GLETDGG
60	GGIETDSG
61	DGIETDSGVDDD
62	GELDSGIETDSG
63	GELQTDGG
64	GSSIETDALG
65	VLLVSEVLDD
66	VVVWSEVVDD
67	GDRVYIHPFHLD
68	GCFTERDDD
69	AAGVAYSEA

70	GGVAYSEGG
71	GALASEIVGG
72	GARAAEIVGG
73	GAEAGEIIGG
74	GASGYGG
75	GPLGLEEAG
76	SGVNLDAGFR
77	DRlVGDDPY
78	GEGPWLEEEG
79	GNFDEIDRGG
80	GRPPGFSPFR
81	GFIFEEEPGG
82	GAAYAAYAG
83	GSAYAATDEGN
84	GNSAAYASGNG
85	GPLGLARDDDD
86	GEPIFFDTWDN
87	EPILCDSWDDD
88	GSWDDDDNGG
89	GLEAIANGG
90	GLQSITHGG
91	GIANDGG
92	GITHDGG
93	GSAPFSFGG
94	GSAPYTYGG
95	GGFAFGISGG
96	GGSLLGSIQGG
97	GGSQTDVVGG
98	GGTVTDVVGG

99	GSIITDVVGG
100	GGHQTDVVGG
101	GSYATDVVGG
102	GSIPFDVVGG
103	GSLTTDVVGG
104	GSFLTDVVGG
105	GSYVHDVVGG
106	GSYVADVVGG
107	GSWEHDVVGG
108	GSWFKDVVGG
109	GSIETDSPGS
110	GGDETDSPGS
111	GSVETDSPGS
112	GSIEPDSLEEGG
113	GSVEPDSLEEGG
114	GGDEVDGAVGGG
115	GGDEVDPAVGGG
116	GSVIQADGWGG
117	GRQRRALEKG
118	GAKIRGQAKG
119	GSGDRMWggG
120	GLGALLRVKRLE
121	GGPQGIWGQ
122	GGPVPLSLVM
123	GGfPRSGGG
124	GGPLGMRGGC
125	GAIEFDSG
126	GGYVADAPDGG

Table S2. Panel of 20 ABNs for *in vivo* administration. Each peptide is chemically bonded to a unique mass-coded glutamic acid-labeled fibroprotein and conjugated to 8-arm PEG nanoscaffolds via maleimide-thiol click chemistry. The ABNs were purified with high performance liquid chromatography, and molecular weights were determined with MALDI-TOF. Uppercase: L-isomer amino acids, lowercase: D-isomer amino acids.

Name	Sequence (N-terminus = NH2, C-terminus = COOH)	MW
A1	e(+3G)(+1V)ndneeGFFs(+4A)r-(ANP)-GGTPFSGQGGC-(PEG8-40kDa)	64710.9
A2	e(+2G)Vndnee(+2G)FFs(+4A)r-(ANP)-GGVAYSEGGC-(PEG8-40kDa)	62528.6
A3	eG(+6V)ndneeG(+10F)(+10F)sAr-(ANP)-GGAAFAGC-(PEG8-40kDa)	59565.2
A4	e(+2G)(+6V)ndneeGFFsAr-(ANP)-GGAIEFDSGC-(PEG8-40kDa)	59710.9
A5	eGVndneeGF(+10F)s(+4A)r-(ANP)-GGSGDRMWggGC-(PEG8-40kDa)	66000.5
A6	e(+2G)Vndnee(+2G)F(+10F)sAr-(ANP)-GGAKIRGQAKGC-(PEG8-40kDa)	63514.2
A7	eG(+6V)ndnee(+3G)(+1F)Fs(+4A)r-(ANP)-GGfPRSGGGC-(PEG8-40kDa)	60210.3
A8	e(+2G)(+6V)ndnee(+3G)(+1F)(+1F)s(+1A)r-(ANP)-GGRQRRALEKGC-(PEG8-40kDa)	64769.9
A9	e(+3G)(+1V)ndnee(+2G)(+10F)Fs(+4A)r-(ANP)-GGLGALLRVKRLEC-(PEG8-40kDa)	66952.9
A10	eGVndnee(+3G)(+1F)Fs(+4A)r-(ANP)-GGYVADAPDGC-(PEG8-40kDa)	64303.8
A11	e(+3G)(+1V)ndneeG(+10F)FsAr-(ANP)-GGELDSGIETDSGC-(PEG8-40kDa)	67842.4
A12	e(+2G)(+6V)ndneeG(+10F)(+1F)s(+1A)r-(ANP)-GGVDQVDGWGC-(PEG8-40kDa)	63864.4
A13	eG(+6V)ndneeG(+10F)Fs(+4A)r-(ANP)-GGPRAAA-Homophe-TSPGC-(PEG8-40kDa)	65764.8
A14	e(+2G)(+6V)ndnee(+3G)(+10F)(+1F)s(+4A)r-(ANP)-GGEEKQRIILGC-(PEG8-40kDa)	66327.0
A15	eGVndnee(+2G)(+10F)(+10F)s(+4A)r-ANP-GGP-(Cha)-G-Cys(Me)-HAGC-(PEG8-40kDa)	60949.4
A16	eG(+6V)ndneeGF(+1F)s(+1A)r-(ANP)-GGRPLGLAGKGC-(PEG8-40kDa)	63575.6
A17	e(+2G)Vndnee(+3G)(+10F)(+1F)s(+4A)r-(ANP)-GGPQGIWGQGC-(PEG8-40kDa)	66158.7
A18	e(+2G)VndneeG(+10F)(+10F)s(+4A)r-(ANP)-GGPLGMRGGC-(PEG8-40kDa)	63497.7
A19	e(+3G)(+1V)ndnee(+2G)(+10F)(+10F)sAr-(ANP)-GGPVGLIGC-(PEG8-40kDa)	60463.1
A20	eGVndneeG(+10F)(+10F)sAr-(ANP)-GGPVPLSLVMC-(PEG8-40kDa)	66457.6

Table S3. Activity zymography probe sequences for *in situ* tissue staining. Uppercase: L-isomer amino acids; lowercase: D-isomer amino acids; U: succinoyl; X: 6-aminohexanoyl.

Substrate Name	Sequence (C-terminus = amide)
A1	UeeeeeeeeeXGTPFSGQGGrrrrrrrrrX-k(5FAM)
A10	UeeeeeeeeeXGGYVADAPDGGrrrrrrrrrX-k(Cy5)
A12	UeeeeeeeeeXGVDQVDGWGGrrrrrrrrrX-k(Cy5)
polyR	rrrrrrrrrX-k(Cy7)

Data S1. (separate file)

Raw data from figures.
